# Temporal and geographical research trends of antimicrobial resistance in wildlife – A bibliometric analysis

**DOI:** 10.1016/j.onehlt.2020.100198

**Published:** 2020-11-21

**Authors:** Rita Tinoco Torres, João Carvalho, Mónica V. Cunha, Emmanuel Serrano, Josman Dantas Palmeira, Carlos Fonseca

**Affiliations:** a Department of Biology & CESAM, University of Aveiro, Portugal; b Centre for Ecology, Evolution and Environmental Changes (cE3c), Faculdade de Ciências, Universidade de Lisboa, 1749-016 Lisbon, Portugal; c Biosystems & Integrative Sciences Institute (BioISI), Faculdade de Ciências, Universidade de Lisboa, 1749-016 Lisbon, Portugal; d Wildlife Ecology and Health Group (WE&H) and Servei d'Ecopatologia de Fauna Salvatge (SEFaS), Departament de Medicina i Cirurgia Animals, Universitat Autònoma de Barcelona, Bellaterra, Barcelona, Spain; e ForestWISE - Collaborative Laboratory for Integrated Forest & Fire Management, Quinta de Prados, 5001-801 Vila Real, Portugal

**Keywords:** AMR, Bibliometric analysis, Wildlife, One health, *E. coli*, Genes

## Abstract

Antimicrobial resistance (AMR) is a complex and global problem. Despite the growing literature on AMR in the medical and veterinary settings, there is still a lack of knowledge on the wildlife compartment. The main aim of this study was to report the global trends in AMR research in wildlife, through a bibliometric study of articles found in the Web of Science database. Search terms were “ANTIMICROBIAL” OR “ANTIBIOTIC” AND “RESISTANT” OR “RESISTANCE” and “WILDLIFE” “MAMMAL” “BIRD” “REPTILE” “FERAL” “FREE RANGE”. A total of 219 articles were obtained, published between 1979 and 2019. A rising interest in the last decades towards this topic becomes evident. During this period, the scientific literature was distributed among several scientific areas, however it became more multidisciplinary in the last years, focusing on the “One Health” paradigm. There was a geographical bias in the research outputs: most published documents were from the United States, followed by Spain, Portugal and the United Kingdom. The most productive institutions in terms of publication number were located in Portugal and Spain. An important level of international collaboration was identified. An analysis of the main keywords showed an overall dominance of “AMR”, “*E. coli”*, “genes”, “prevalence”, “bacteria”, “*Salmonella* spp.” and “wild birds”. This is the first study providing a global overview of the spatial and temporal trends of research related to AMR in wildlife. Given the growth tendency over the last years, it is envisaged that scientific production will expand in the future. In addition to offering a broad view of the existing research trends, this study identifies research gaps both in terms of geographical incidence and in relation to unexplored subtopics. Unearthing scientific areas that should be explored in the future is key to designing new strategic research agendas in AMR research in wildlife and to inform funding programs.

## Highlights

• First study providing a global overview of the spatial and temporal trends of research related to AMR in wildlife.
• Rising interest on AMR research on wildlife worldwide but there is a spatial and taxonomic bias in the research outputs.
• This study highlights poorly surveyed countries, where intensified sampling efforts are crucial.
• Research trends and scientific areas are unearthedthat should be invested in and explored in the future.

## Graphical abstract

**Figure:**
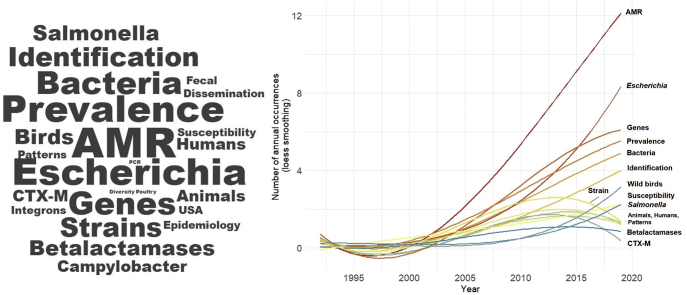

## 1 Introduction

Antimicrobials are essential for the treatment of bacterial infections in humans and animals and have revolutionized human healthcare practices worldwide. Penicillin, for instance, lowered mortality linked with pneumococcal pneumonia from 20 to 40% to 5% and mortality from pneumococcal bacteremia from 50 to 80% to 18–20% [1,23]. Bacterial resistance to antimicrobials, however, quickly became a substantial clinical problem threatening the advances of the previous decades [4] and posing a significant threat to public health. When a microorganism that was susceptible to an antibiotic is no longer sensitive due to the acquisition of resistance determinants, antibiotics become less effective and treatment options are limited. This acquired resistance phenotype contrasts with natural resistance presented by several bacteria, which has existed for millions of years, and is an evolutionary consequence of microbial competition in their ecological niches [5]. Bacteria acquire resistance through mutations and horizontal gene transfer of resistance determinants. Direct inactivation of antibiotics (*e.g.*, by β-lactamases), modification (*i.e.*, mutation) of cellular targets and modification of cell wall, are examples of resistance strategies/mechanisms that microorganisms employ [6]. Mutation and mobilization of genes encoding resistance mechanisms, as well as adaptive resistance phenotypes, are fostered by the same factors that promote antibiotic usage, particularly prolonged, cumulative, low-level exposure, including antibiotic overuse, demographic changes associated with urbanization and poor sanitation, discharge of antibiotic residues through environmental wasting and biocide use in livestock production [1,2,7]. Still, antibiotic consumption and overuse are considered the primary drivers of AMR [8] and a substantial part of the resistance burden in humans is attributable to antimicrobial use in livestock production, primarily for disease prevention and growth promotion purposes [5,7]. For example, antimicrobials used in livestock are expected to account for approximately 80% of the U.S.A. annual antimicrobials consumption [9] and 73% globally [10].

AMR is now recognized as a complex, multi-layered global problem, that extends beyond national and animal borders, threatening human, animal and environmental health [[11], [12], [13]]. Various authors have strongly encouraged a holistic and multidisciplinary “One Health” approach to tackle AMR, while stressing that the increasing incidence of AMR in humans and livestock has been linked to the emergence of AMR in wildlife [14,15]. Despite a large, and growing, literature on AMR in medical and veterinary settings, there is still a dearth of research on the complex transmission dynamics of AMR in the environmental and wildlife compartments [16], even though the range, distribution and number of wild species (only birds [17] and mammals [18] is around 600 times higher than livestock (40 species and 4500 breeds). Several studies have reported wildlife species as potentially important reservoirs of resistant microorganisms and resistance genes [19,20]. For example, *Escherichia coli* isolates producing extended spectrum beta-lactamases (ESBL) have been isolated from wild boar (*Sus scrofa*) in several European countries [[21], [22], [23]], putting at stake the efficacy of beta-lactam antibiotics (*e.g.* penicillin), which are among the most important class of antimicrobial agents used in human and veterinary medicine.

So, there is an urgent missing link, that upon revelation will contribute to the understanding of the origins and roles of antibiotic resistance genes in the gut microbiota of wildlife and the complex transmission dynamics of the underlying determinants in the environmental setting [15,16]. Howbeit, AMR is deemed as one of the major public health concerns of the 21st century [3,24], knowledge concerning AMR bacteria circulating in wildlife is currently limited, although available literature suggests that this wild compartment could provide important insights into AMR emergence and persistence [15,16]. Theoretically, wild animals are not treated with antibiotics, but their association, both direct and indirect, with humans, livestock, domestic animals or humanized-environments, their ability to easily move across environmental gradients of humanization (from pristine – natural – agroforestry – to highly humanized scenarios), can enhance their contact with selective agents, with commensals from humans and other species, as well as with resistant bacteria. This contact is considered to promote adaptation mechanisms of commensal bacteria and horizontal transfer of resistance genes within the bacterial community of wildlife. Additionally, some of these species (*e.g.* wild ungulates such as wild boar, among others) are emerging as source of foodborne pathogens in humans due to the manipulation and consumption of game meat [[25], [26], [27], [28]]. Altogether, AMR research has to assume a multidisciplinary dimension crossing fields such as microbiology, genomics, environmental science, ecology, agriculture, pharmaceutical industry, synthetic biology, biotechnology and health sciences [29]. Neglecting the dialog across different disciplines will hamper our ability to detect, and thus control, the increasing complexities of the factors involved in AMR dynamics.

An analysis that could depict the relevance of AMR in wildlife would be of value, not only to offer a baseline to identify hotspots of AMR, from which research gaps could be identified and launching a starting point not only to academic researchers but to various stakeholders involved in the topic. The analysis of research trends through bibliometric studies is receiving considerable attention, as they provide valuable information on scientific research and its progression in a specific field of research [30]. Such analysis allows mapping the structure and accumulation of scientific knowledge in specific fields, allowing the assessment of the evolution of specific disciplines [31] by categorizing descriptors such as citations, years, author affiliations, keywords, countries, publication categories, among others. Previous bibliometric studies on AMR were related to drug-resistance in specific diseases and bacteria [[32], [33], [34], [35], [36], [37], [38]] disease surveillance programs [38] as well as social impact [39].

This study exposes the gaps in the literature relating to the role of wildlife as drivers for the spread of AMR bacteria, by (1) providing a global overview of the spatial and temporal trends of reported scientific knowledge on antimicrobial resistance in wildlife and (2) identifying relevant research gaps both in terms of geographical incidence and also in relation to the subtopics that should be addressed. To deliver such information, peer-reviewed publications of AMR in wildlife were retrieved from the Web of Science, systematized and examined to illustrate the trends and evolutions on this topic.

## 2 Material and methods

### 2.1 Data collection

#### 2.1.1 Data collection

A systematic literature review was performed using a rigorous search strategy in the online version of the Science Citation Index Expanded (SCI-EXPANDED) from the Web of Science (WoS) database (http://www.isiknowledge.com), which is one of the largest and comprehensive bibliographic databases covering multidisciplinary areas. WoS was chosen as it is the oldest citation database, including records that go back to 1900 [49]. Search results were delimited based on the following Boolean query executed within a single search (conducted in November 2019). No time and geographical location restrictions were placed on these searches, and only those published in English were retrieved. The searches were last updated on 26th November 2019. The search strategy consisted of compiling three search strings, one for each category (antimicrobial resistance and wildlife) and combining these by the Boolean operator “AND” to obtain only the intersection. Specifically, we used the following Boolean search statement: #1 “antimicrobial resistance”: “ANTIMICROBIAL” OR “ANTIBIOTIC” AND “RESISTANT” OR “RESISTANCE” and #2 “wildlife”: “WILDLIFE” “MAMMAL” “BIRD” “REPTILE” “FERAL” “FREE RANGE” and the interception consisted in #1 AND #2. The search was made to the whole data series available, that is, in the last 40 years, from 1979 to 2019. Articles originating from England, Scotland, Northern Ireland, and Wales were reclassified as being from the United Kingdom (UK).

#### 2.1.2 Data analysis

**Fig. 1:**
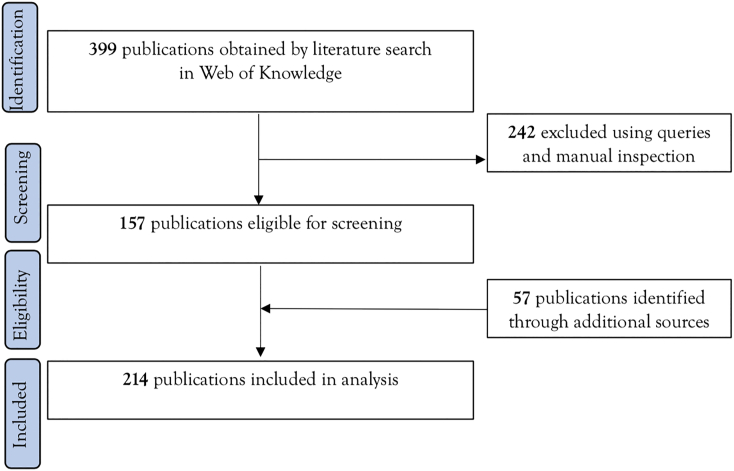
Scoping review flowchart of the dataset selection process. The PRISMA flow diagram of the search strategy, study selection and data management procedure.

Results for all articles were imported into a bibliographic referencing tool and assessed for relevance, removing articles that did not contain information relating to AMR in wildlife. All query results were verified manually before excluding duplicates (Fig. 1 – flowing chart). All publications were included with the following variables extracted: publication date, subject category, document type, author, organization of origin, funding agency, language, country of origin, title, abstract, and keyword. Once the manuscripts had been obtained, the study of research trends was carried out through the analysis of scientific production per year, type of document, distribution in subject categories and source, publication distribution by countries and institutions, and an analysis of index keywords. The bibliometric analysis was performed on the full search results using the *bibliometrix* package in R [50].

## 3 Results and discussion

The present study outlines the bibliometric indicators of the reported scientific research related to AMR in wildlife during the timeframe from 1979 to 2019 (40 years). The initial 399 records were transferred to Mendeley: duplicates and not relevant publications (*e.g.*, publications related to the environmental, human, livestock and domestic setting) were removed. After the initial screening, a total of 162 publications were considered. We completed the research selection with additional publications (*n* = 57) cited in review papers [19,20]. Overall, 219 publications were included in the analysis (Fig. 1) (supplementary material).

### 3.1 Temporal evolution of scientific research

**Fig. 2:**
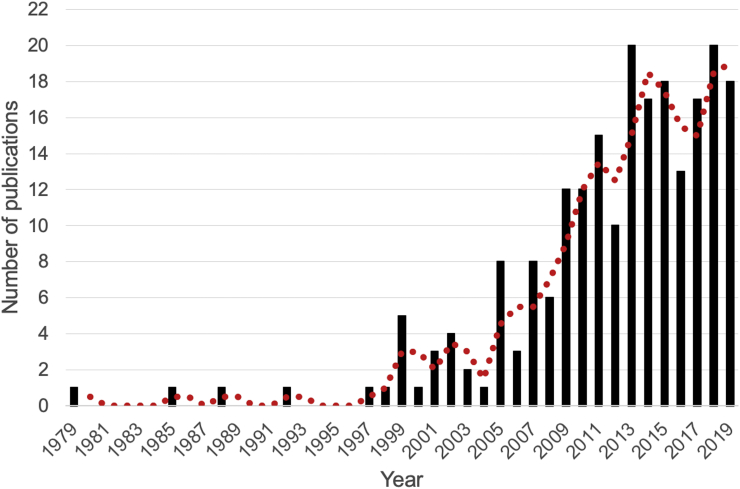
Evolution of published papers on AMR in wildlife from 1979 to 2019.

Publications dated from 1979 to 2019 and, overall, the temporal trend in publication on antimicrobial resistance in wildlife shows a growth in the number of documents published per year, with an annual percentage growth rate of 7,2% (Fig. 2). The global evolution of literature can be split into two periods, exhibiting kind of a diauxic growth: from 1979 to 2008, the scientific literature increased slowly (only 47 publications). However, from 2009 to 2019, the growth was steady and swift; 78% of the research papers were published in the last ten years. This indicates that this research topic has attracted particular interest (and perhaps funding) in the last decade, likely a reflex of the increase of global importance of the AMR subject as more countries and institutions began to devote themselves to this topic. The maximum number of documents on AMR in wildlife was published in 2018, with a total of 20 publications, but 2019 should follow the same trend as the database was last updated in November, accounting already 17 publications. This rejuvenated interest in AMR research in wildlife can be attributed to the fact that three of the most cited research in AMR in wildlife were published in previous years and in relevant journals (Table 1). Additionally, and perhaps more importantly, since the first studies regarding antibiotic resistance in wildlife, a discussion was triggered whether resistance in wildlife could or not be related with human antibiotic use [40,41]. High prevalence of bacteria with antibiotic resistance was detected by [40] from wild rodents occurring in rural areas in Wirral, northwest England, in areas with absent or minimum levels of released antibiotics. Such fact led [40] to claim that the found prevalence was not directly a result of anthropogenic impact and that antibiotic use restrictions would have marginal effect of wildlife reservoirs. Contrastingly [41], described almost no resistance in bacteria recovered from moose, deer and voles in pristine areas of Finland. These two seminal papers brought into debate the effects of human proximity, highlighting the importance of understanding the role of wildlife in the ecology of antibiotic resistance. Since then, research has been focused on untangling the routes of transmission of AMR between humans and wildlife, reinforcing the idea that the same antimicrobial resistance patterns co-occur in wildlife, livestock and human populations. For example, beta-lactamases are now frequently found in bacterial isolates from wildlife [42], particularly birds and mammals [43,44], as well as in livestock and environmental samples.

**Table 1:** Top papers per citations, ranked by total citations.

	Paper	TC	TC per year
1st	Sayah R, 2005, *Appl Environ Microbiol*	207	13.80
2nd	Hagedorn C, 1999, *Appl Environ Microbiol*	179	8.52
3rd	Gilliver M, 1999, Nature	162	7.71
4th	Bryan A, 2004, *Appl Environ Microbiol*	145	9.06
5th	Souza V, 1999, *Appl Environ Microbiol*	136	6.48
6th	Kozak G, 2009, *Appl Environ Microbiol*	130	11.82
7th	Skurnik D, 2006, *J Antimicrob Chemother*	116	8.29
8th	Costa D, 2006, *J Antimicrob Chemother*	107	7.64
9th	Rwego I, 2008, *Conserv Biol*	93	7.75
10th	Literak I, 2010, *Appl Environ Microbiol*	90	9.00

#### 3.1.1 Most cited papers

The top 10 papers per citations related to the role of wildlife in AMR are listed in Table 1. The most cited paper was published in 2005 by Sayah et al. in Applied Environmental Microbiology (*n* = 207), by researchers from the University of Michigan and University of Maryland, U.S.A. The paper compares antimicrobial agent resistance profiles of normal gut microbiota from samples of domestic livestock, poultry, pets, wildlife, and humans in the same geographic region, suggesting that the rate of *E. coli* recovery may be different for different species. The second most cited paper (*n* = 179), was also published in Applied Environmental Microbiology in 1999 by Hagedorn et al., by researchers from Department of Crop and Soil Environmental Sciences (Virginia Polytechnic Institute) and State University (Virginia), U.S.A. The paper identifies sources of fecal pollution of a watershed in rural Virginia, from a variety of sources including humans, livestock (cattle, chickens) and wildlife (deer, geese and ducks). The third most cited paper (*n* = 162) was published in Nature, by Gilliver et al., where authors showed that antimicrobial resistance was prevalent (90%) in wildlife species (*e.g.*, wild rodents) even in the absence of direct exposure to antibiotics, highlighting that the origin of AMR persistence and dissemination is not always known.

#### 3.1.2 Distribution of publications in subject categories

**Fig. 3:**
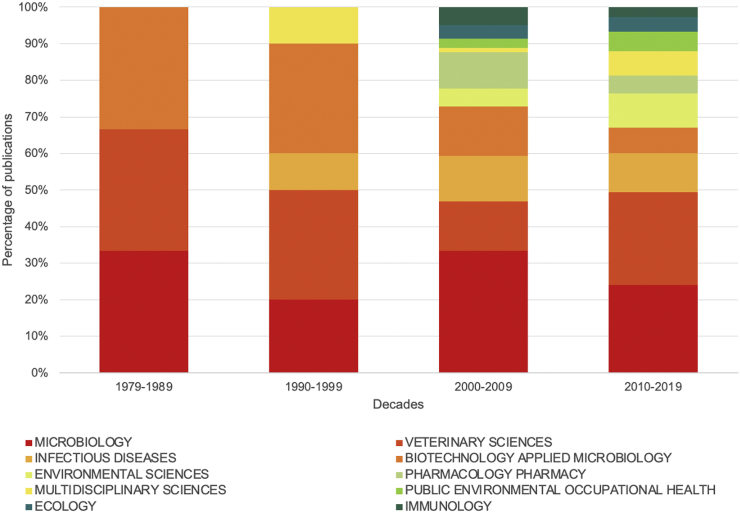
Evolution of published papers in subject categories, from 1979 to 2019.

From 1979 to 2019, the scientific literature was distributed among a broad range of scientific areas (WoS subject categories): 28 subject categories in total, with Microbiology (40%) and Veterinary Sciences (33%) as the most targeted areas. The results suggest that these two areas remained a top priority among the various topics being explored in AMR research in wildlife. From 1979 to 1989, microbiology, veterinary sciences and infectious diseases held primacy in relation to the other areas (Fig. 3); however, since 2000 the number of articles in environmental sciences and ecology have gained in importance. It is interesting to note that during the 1979–2019 period this topic became more multidisciplinary, which indicates a change of the spotlight of AMR studies to an emphasis on the “One Health” framework, reflecting the inception of the “One Health” paradigm and possibly the awareness of researchers in the veterinary field for conducting studies on AMR-related topics. During this 40 year period, research shifted from Microbiology subject area into a multidisciplinary area, stressing that the key factor for this increment in the number of research/publications is an investment in multidisciplinary research. Interestingly, Ecology and Environmental Sciences subject areas have been well represented in the last decade. This goes in line with several authors highlighting that the rising threat of AMR requires a holistic and multidisciplinary approach [12]. We are now in an exciting and turning point where One Health can lead to a paradigm shift that will set the foundation to a more integrative and multidisciplinary action for addressing AMR challenges.

#### 3.1.3 Publication distribution by country and collaborations among countries

**Fig. 4:**
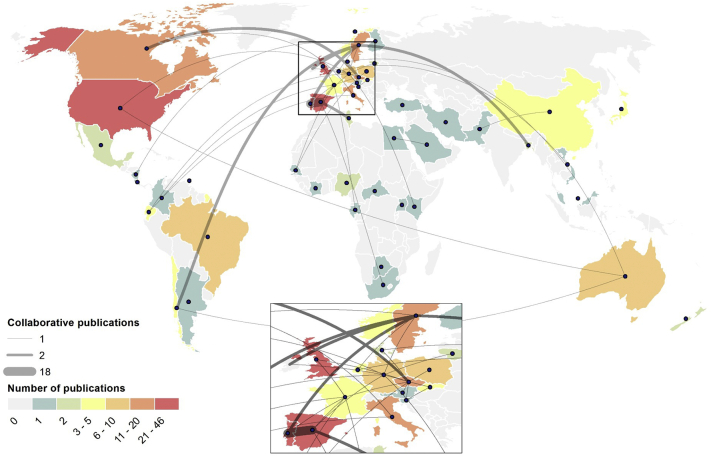
Global scientific production and international collaboration on AMR in wildlife.

Scientific production at the global level is presented in Fig. 4, where it becomes evident that industrialized countries were the most productive countries in terms of research outputs. A total of 50 countries published research in AMR in wildlife over the last 40 years. Of these, five countries contributed to approximately 72% of research publications total. United States, as well as the concentration of research in European countries, especially Spain, Portugal, United Kingdom and Sweden, stand out. In addition, Czech Republic and Italy in Europe, are prominent, as well as Canada and Australia. These results are not surprising and the dominance of these countries is probably related to their economic development and substantial amount of financial support to researchers, which has already been linked to overall academic output [45]. The dominance of Portugal, Spain and Sweden is likely related to some prolific authors developing their interest. Fig. 4 show regions that are poorly surveyed and where intensified sampling efforts could be most valuable, namely Asia, Africa and South America. A special focus has to be devoted to these countries as human populations are growing and landscapes are being transformed rapidly [46]. showed that overall antibiotic consumption between 2000 and 2015 has increased by 65%, and in developing countries it has been meeting, or even exceeding, the levels observed in developed countries. Additionally [10], mapped resistance in livestock, shows that the largest hotspots of AMR in these animals are in China and India, with emerging countries such as Brazil and Kenya, all countries where research in AMR in wildlife has been residual or absent. The increase in meat production and demand, and the shift in livestock production systems in developing countries, stresses the importance to implement actions to prevent further aggravation of the AMR problem. This can be done by increasing collaborative research within this topic with countries where the laboratory and analytical infrastructures are already implemented but also by increased funding availability to increased infrastructures and qualified researchers in these countries, which will obviously translate into an increase in publications.

Interestingly, U.S.A. was also the country with a higher number of citations (total citations 1211), however it was the Czech Republic which scored the highest in the average article citation (39) (Table 2). It is important to stress that the number of citations is not a straightforward indicator of a paper quality but rather a measure of its impact among peers and/or visibility.

**Table 2:** Total citations per Country.

	Country	Total Citations	Average Article Citations
1st	U.S.A.	1211	32.730
2nd	Czech Republic	509	39.154
3rd	Spain	508	23.091
4th	Portugal	401	18.227
5th	United Kingdom	342	34.200

Two countries stand out with intense cooperation among themselves: Portugal and Spain in the Iberian Peninsula. This region has several scientific and technological activities which aim to intensify and consolidate strong scientific collaboration, apart from the cultural and language link [47]. Additionally, among the top 5 of the most productive authors, four are from the Iberian Peninsula (Table 2). Consistent with observations in other research fields, a small group of prolific authors contributed to a significant share of publications. For example, the top 5 authors, produced 41% of the total publications. Considering the number of publications the most productive authors in AMR research in wildlife were P. Poeta with 22 publications (10%), followed by C. Torres with 20 papers (9%), G. Igrejas 18 (8%), B. Olsen with 14 (7%), and A. Gonçalves with 13 (6%).

Spain, Germany, Sweden and France maintained active collaborations (Fig. 4). The overview of publications that include international collaboration is a good indicator that research in this topic is becoming more internationally connected, a fact that can be observed in the map of global collaboration. Furthermore, international collaboration also demonstrates the importance of large collaborative networks to tackle AMR in wildlife, where ecological factors (*e.g.*, migratory behavior) contribute to the dissemination of resistance genes [15]. Overall, such information is valuable to discover new places where new work should start or where to build up some collaborations.

#### 3.1.4 Most productive institutions

The top 10 institutions were ranked by the number of articles. Among the 250 institutions that participated in AMR research in wildlife, the University of Trás-os-Montes and Alto Douro (Portugal) led institutional productivity with 32 (15%) papers, followed by University of La Rioja (Spain) with 18 (8%), the University of Uppsala (Sweden) with 15 (7%), the University of Linnaeus (Sweden), University of Veterinary Pharmaceutical Sciences Bnro (Czech Republic), both with 14 (7%), University of Guelph (Canada) with 12 (6%), the Public Health Agency of Canada with 9 (5%), Autonomous University of Barcelona with 9 (4%), Kalmar City Hospital (Sweden) with 9 (4%) and University of Porto (Portugal) with 7 (3%).

#### 3.1.5 Analysis of keywords

**Fig. 5:**
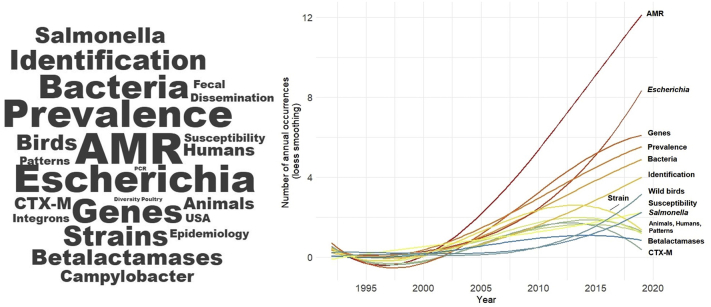
Word cloud based on the main keywords related to worldwide research focused on AMR in wildlife for the 1979 to 2019 period (left) and its evolution (right).

**Fig. 6:**
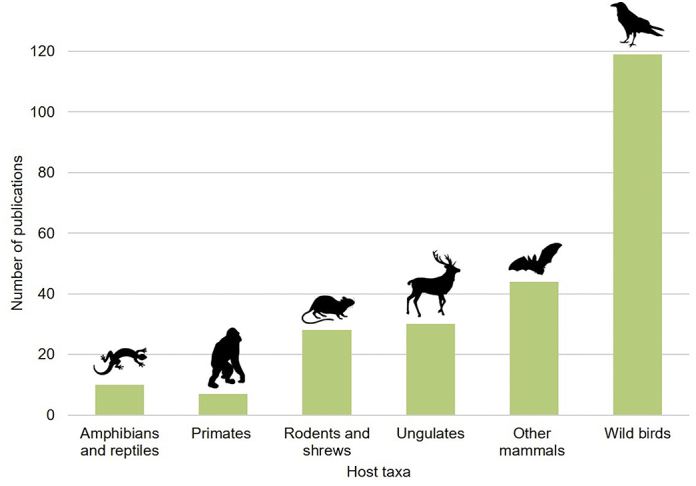
Number of publications by host taxon group.

Evaluation of the keywords in a publication is useful to provide a detailed picture of a publication's theme, reflecting the research hotspots in the discipline fields, therefore helping researchers to explore dominant research topics. Our analysis to the analyzed publications keyword showed that the most common keywords were AMR, *E. coli*, genes, prevalence, bacteria, *Salmonella* spp. and wild birds (Fig. 5). This emphasizes that studies have focused in determining the antimicrobial susceptibility of specific indicator bacteria such as *Escherichia coli* and *Salmonella* spp. The choice of these bacterial species is mostly linked to their relevance as human foodborne pathogens. *E. coli* is also part of the mammals' gut microbiota and can easily be disseminated in different ecosystems, facilitating the direct comparison of resistance phenotypes in distinct environments and host animals. Most studies have focused on searching for specific bacteria rather than search for the whole bacterial community. This is obviously a limitation and future research should concentrate on a wider range of bacteria groups. It also stresses a taxonomic bias regarding the taxa hosts, as mostly wild birds have been used as model species to determine antimicrobial resistance profiles (Fig. 6), probably due to their wide migration routes but also their suitability to explore anthropogenic gradients, from natural to humanized (*e.g.* landfills) environments. In fact, migratory birds can acquire antibiotic resistance during their migratory stop-overs and can therefore act as a reservoir and long-distance disperser of antibiotic resistant bacteria. Small mammals have also been widely use as sentinel species most likely due to their density in the environment, high levels of interaction with humans and domestic animals and their proximity, and use, of anthropogenic waste. However, as humans transform landscapes the contact with wildlife concomitant increases, and *circa* of 70% of the majority of emerging infectious diseases in humans arise from wildlife reservoirs [48]. Several mammal species due to their ecology (omnivorous, synantropic) or to their close association with humans (are hunted, consumed, wide distribution ranges, etc) could serve as key epidemiological and be defined as priority species for surveillance and used for target monitoring and designing proactive management programs, such as the wild boar [28]. Future steps should aim to reduce this taxonomic bias and evaluate the potential of various mammal species as AMR indicators in the environment. This would be a valid tool to inform public health agencies as a mean to develop, and implement, tailored mitigation strategies.

#### 3.1.6 Limitations

It is important to acknowledge that bibliometric studies carry a number of limitations. Firstly, we only focused on articles that have been published in academic journals indexed in Web of Science, excluding the amount of work that may have been published in other formats (*e.g.* books, reports, and national journals). Publications that did not include the used search terms in the title might have been left out of our analysis. Nevertheless, our results reflect perceived interest by the scientific community. In addition, this analysis was restricted to international journals in English, therefore a linguistic bias may also exist. Additionally, the number of publications and citations should be noted as a proxy of the scientific relevance of a subject and not of the quality of the underlying work and publication itself. Nevertheless, we believe that our findings offer a valid representation within this research field at a global level.

## 4 Conclusion

This study provides an overview of AMR research in wildlife on worldwide scale, reporting valuable information related to annual publication numbers, categories, institutions, countries, and researchers. Important features and trends in science and performance during the period for 1979 to 2019 have been unearthed. All of the analyzed bibliometric variables in this study revealed solid growth within this research field, both in terms of increasing scientific production and research collaboration. Increasingly, more researchers, institutions and countries got involved in AMR research in wildlife over this period. However, research output was distributed unevenly over all countries, with the industrialized countries being more productive and owing more collaborations among them and with other countries with lower funding availability and research tradition in this area. While most research was focused on the Microbiology and the Veterinary Sciences subject categories in the initial publications, during the analyzed period this topic became more multidisciplinary likely due to the recognized of the “One Health” framework in AMR. Our findings show the value of bibliometric methods to illustrate global research trends of AMR research in wildlife. Thus, this study provides a helpful reference for academics, veterinarians and policy decision makers. As research in AMR focused on wildlife is still in its infancy, our findings provide a ‘snapshot’ of this field at an early stage of its development. But the study of AMR in wildlife, only makes sense in the light of landscape ecology. Therefore, future studies must overlap infectious disease ecology, landscape ecology, and microbiology, to infer emergence, transmission and identify environmental drivers of AMR spread across space and between species. Such approach will significantly contribute to disclose the dynamics of AMR in the wildlife interface by identifying populations at risk, mapping high-risk areas and, consequently, by directing surveillance programs and designing proactive management actions.

## Ethical statement

Not applicable.

## Funding statement

This work was supported by the project EcoARUn: POCI-01-0145-FEDER-030310- funded by FEDER, through COMPETE2020 - Programa Operacional Competitividade e Internacionalização (POCI), and by national funds (OE), through FCT/MCTES.

## Author statement

The idea for the study was devised by RTT and MVC. RTT wrote the manuscript. RTT and JC analyzed the data. All authors gave intellectual input and critically revised the manuscript.

## Declaration of Competing Interest

The authors declare no conflict of interest.

## Appendix A Supplementary data

List of the publications. Image 1
