# Supplementary material for: Temporal and geographical research trends of antimicrobial resistance in wildlife – A bibliometric analysis
Source: One Health. 2020 Nov 21;11:100198. doi: 10.1016/j.onehlt.2020.100198 (PMC7702190; doi:10.1016/j.onehlt.2020.100198)
Supplement: Supplementary file 1 — List of the publications. [file mmc1.docx]

Abo-Amer, A. E., and Shobrak, M. Y. (2015). Antibiotic Resistance and Molecular Characterization of Enterobacter cancerogenus Isolated from Wild Birds in Taif Province, Saudi Arabia. *THAI J. Vet. Med.* 45, 101–111.

Adesiyun, A. A., Seepersadsingh, N., Inder, L., and Caesar, K. (1998). Some bacterial enteropathogens in wildlife and racing pigeons from Trinidad. *J. Wildl. Dis.* 34, 73–80.

Adesiyun, A., and Downes, M. (1999). Prevalence of antimicrobial resistance and enteropathogenic serogroups in Escherichia coli isolates from wildlife in Trinidad and Tobago. *Vet. Arh.* 69, 335–347.

Agnew, A., Wang, J., Fanning, S., Bearhop, S., and McMahon, B. J. (2016). Insights into antimicrobial resistance among long distance migratory East Canadian High Arctic light-bellied Brent geese (Branta bernicla hrota). *Ir. Vet. J.* 69. doi:10.1186/s13620-016-0072-7.

Ahlstrom, C. A., Bonnedahl, J., Woksepp, H., Hernandez, J., Reed, J. A., Tibbitts, L., et al. (2019). Satellite tracking of gulls and genomic characterization of faecal bacteria reveals environmentally mediated acquisition and dispersal of antimicrobial-resistant Escherichia coli on the Kenai Peninsula, Alaska. *Mol. Ecol.* 28, 2531–2545. doi:10.1111/mec.15101.

Aksomaitiene, J., Ramonaite, S., Tamuleviciene, E., Novoslavskij, A., Alter, T., and Malakauskas, M. (2019). Overlap of Antibiotic Resistant Campylobacter jejuni MLST Genotypes Isolated From Humans, Broiler Products, Dairy Cattle and Wild Birds in Lithuania. *Front. Microbiol.* 10. doi:10.3389/fmicb.2019.01377.

Albrechtova, K., Papousek, I., De Nys, H., Pauly, M., Anoh, E., Mossoun, A., et al. (2014). Low Rates of Antimicrobial-Resistant Enterobacteriaceae in Wildlife in Tai National Park, Cote d’Ivoire, Surrounded by Villages with High Prevalence of Multiresistant ESBL-Producing Escherichia coli in People and Domestic Animals. *PLoS One* 9. doi:10.1371/journal.pone.0113548.

Alhaji, N. B., Haruna, A. E., Muhammad, B., Lawan, M. K., and Isola, T. O. (2018). Antimicrobials usage assessments in commercial poultry and local birds in North-central Nigeria: Associated pathways and factors for resistance Check to emergence and spread. *Prev. Vet. Med.* 154, 139–147. doi:10.1016/j.prevetmed.2018.04.001.

Allen, S. E., Boerlin, P., Janecko, N., Lumsden, J. S., Barker, I. K., Pearl, D. L., et al. (2011). Antimicrobial Resistance in Generic Escherichia coli Isolates from Wild Small Mammals Living in Swine Farm, Residential, Landfill, and Natural Environments in Southern Ontario, Canada. *Appl. Environ. Microbiol.* 77, 882–888. doi:10.1128/AEM.01111-10.

Allen, S. E., Janecko, N., Pearl, D. L., Boerlin, P., Reid-Smith, R. J., and Jardine, C. M. (2013). Comparison of Escherichia coli Recovery and Antimicrobial Resistance in Cecal, Colon, and Fecal Samples Collected from Wild House Mice (Mus musculus). *J. Wildl. Dis.* 49, 432–436. doi:10.7589/2012-05-142.

Alonso, C. A., Gonzalez-Barrio, D., Tenorio, C., Ruiz-Fons, F., and Torres, C. (2016). Antimicrobial resistance in faecal Escherichia coli isolates from farmed red deer and wild small mammals. Detection of a multiresistant E-coli producing extended-spectrum beta-lactamase. *Comp. Immunol. Microbiol. Infect. Dis.* 45, 34–39. doi:10.1016/j.cimid.2016.02.003.

Alroy, K., and Ellis, J. C. (2011). PILOT STUDY OF ANTIMICROBIAL-RESISTANT ESCHERICHIA COLI IN HERRING GULLS (LARUS ARGENTATUS) AND WASTEWATER IN THE NORTHEASTERN UNITED STATES. *J. ZOO Wildl. Med.* 42, 160–163.

Anderson, J. F., Parrish, T. D., Akhtar, M., Zurek, L., and Hirt, H. (2008). Antibiotic resistance of enterococci in American bison (Bison bison) from a nature preserve compared to that of enterococci in pastured cattle. *Appl. Environ. Microbiol.* 74, 1726–1730. doi:10.1128/AEM.02164-07.

Ardiles-Villegas, K., Gonzalez-Acuna, D., Waldenstrom, J., Olsen, B., and Hernandez, J. (2011). Antibiotic Resistance Patterns in Fecal Bacteria Isolated from Christmas Shearwater (Puffinus nativitatis) and Masked Booby (Sula dactylatra) at Remote Easter Island. *AVIAN Dis.* 55, 486–489.

Arnold, K. E., Williams, N. J., and Bennett, M. (2016). `Disperse abroad in the land’: the role of wildlife in the dissemination of antimicrobial resistance. *Biol. Lett.* 12. doi:10.1098/rsbl.2016.0137.

Asakura, H., Sakata, J., Nakamura, H., Yamamoto, S., and Murakami, S. (2019). Phylogenetic Diversity and Antimicrobial Resistance of Campylobacter coli from Humans and Animals in Japan. *MICROBES Environ.* 34, 146–154. doi:10.1264/jsme2.ME18115.

Barksdale, S. M., Hrifko, E. J., and van Hoek, M. L. (2017). Cathelicidin antimicrobial peptide from Alligator mississippiensis has antibacterial activity against multi-drug resistant Acinetobacter baumanii and Klebsiella pneumoniae. *Dev. Comp. Immunol.* 70, 135–144. doi:10.1016/j.dci.2017.01.011.

Ben Yahia, H., Chairat, S., Hamdi, N., Gharsa, H., Ben Sallem, R., Ceballos, S., et al. (2018). Antimicrobial resistance and genetic lineages of faecal enterococci of wild birds: Emergence of vanA and vanB2 harbouring Enterococcus faecalis. *Int. J. Antimicrob. Agents* 52, 936–941. doi:10.1016/j.ijantimicag.2018.05.005.

Benavides, J. A., Godreuil, S., Bodenham, R., Ratiarison, S., Devos, C., Petretto, M.-O., et al. (2012). No Evidence for Transmission of Antibiotic-Resistant Escherichia coli Strains from Humans to Wild Western Lowland Gorillas in Lope National Park, Gabon. *Appl. Environ. Microbiol.* 78, 4281–4287. doi:10.1128/AEM.07593-11.

Blackburn, J. K., Mitchell, M. A., Blackburn, M.-C. H., Curtis, A., and Thompson, B. A. (2010). EVIDENCE OF ANTIBIOTIC RESISTANCE IN FREE-SWIMMING, TOP-LEVEL MARINE PREDATORY FISHES. *J. ZOO Wildl. Med.* 41, 7–16. doi:10.1638/2007-0061.1.

Blanco-Pena, K., Esperon, F., Torres-Mejia, A. M., de la Torre, A., de la Cruz, E., and Jimenez-Soto, M. (2017). Antimicrobial Resistance Genes in Pigeons from Public Parks in Costa Rica. *Zoonoses Public Health* 64, e23–e30. doi:10.1111/zph.12340.

Blyton, M. D. J., Pi, H., Vangchhia, B., Abraham, S., Trott, D. J., Johnson, J. R., et al. (2015). Genetic structure and antimicrobial resistance of Escherichia coli and cryptic clades in birds with diverse human associations. *Appl. Environ. Microbiol.* 81, 5123–5133. doi:10.1128/AEM.00861-15.

Bondo, K. J., Pearl, D. L., Janecko, N., Boerlin, P., Reid-Smith, R. J., Parmley, J., et al. (2016). Epidemiology of Antimicrobial Resistance in Escherichia coli Isolates from Raccoons (Procyon lotor) and the Environment on Swine Farms and Conservation Areas in Southern Ontario. *PLoS One* 11, e0165303.

Bonnedahl, J., Drobni, P., Johansson, A., Hernandez, J., Melhus, A., Stedt, J., et al. (2010). Characterization, and comparison, of human clinical and black-headed gull (Larus ridibundus) extended-spectrum beta-lactamase-producing bacterial isolates from Kalmar, on the southeast coast of Sweden. *J. Antimicrob. Chemother.* 65, 1939–1944. doi:10.1093/jac/dkq222.

Bonnedahl, J., Hernandez, J., Stedt, J., Waldenstrom, J., Olsen, B., and Drobni, M. (2014). Extended-Spectrum beta-Lactamases in Escherichia coli and Klebsiella pneumoniae in Gulls, Alaska, USA. *Emerg. Infect. Dis.* 20, 897–899. doi:10.3201/eid2005.130325.

Bonnedahl, J., Stedt, J., Waldenstrom, J., Svensson, L., Drobni, M., and Olsen, B. (2015). Comparison of Extended-Spectrum beta-Lactamase (ESBL) CTX-M Genotypes in Franklin Gulls from Canada and Chile. *PLoS One* 10. doi:10.1371/journal.pone.0141315.

Botti, V., Navillod, F. V., Domenis, L., Orusa, R., Pepe, E., Robetto, S., et al. (2013). Salmonella spp. and antibiotic-resistant strains in wild mammals and birds in north-western Italy from 2002 to 2010. *Vet. Ital.* 49, 195–202.

Bryan, A., Shapir, N., and Sadowsky, M. J. (2004). Frequency and distribution of tetracycline resistance genes in genetically diverse, nonselected, and nonclinical Escherichia coli strains, isolated from diverse human and animal sources. *Appl. Environ. Microbiol.* 70, 2503–2507. doi:10.1128/AEM.70.4.2503-2507.2004.

Cabrita, J., Rodrigues, J., Bragança, F., Morgado, C., Pires, I., and Gon??alves, A. P. (1992). Prevalence, biotypes, plasmid profile and antimicrobial resistance of Campylobacter isolated from wild and domestic animals from Northeast Portugal. *J. Appl. Bacteriol.* 73, 279–285. doi:10.1111/j.1365-2672.1992.tb04978.x.

Caleja, C., de Toro, M., Goncalves, A., Themudo, P., Vieira-Pinto, M., Monteiro, D., et al. (2011). Antimicrobial resistance and class I integrons in Salmonella enterica isolates from wild boars and Bisaro pigs. *Int. Microbiol.* 14, 19–24. doi:10.2436/20.1501.01.131.

Camarda, A., Circella, E., Pennelli, D., Madio, A., Bruni, G., Lagrasta, V., et al. (2006). Wild birds as bioloigical indicators of environmental pollution: biotyping and antimicrobial resistance patterns of Escherichia coli isolated from Audouin’s gulls (Larus audouinii) living in the Bay of Gallipoli (Italy). *Ital. J. Anim. Sci.* 5, 287–290.

Carlson, J. C., Hyatt, D. R., Ellis, J. W., Pipkin, D. R., Mangan, A. M., Russell, M., et al. (2015). Mechanisms of antimicrobial resistant Salmonella enterica transmission associated with starling-livestock interactions. *Vet. Microbiol.* 179, 60–68. doi:10.1016/j.vetmic.2015.04.009.

Carroll, D., Wang, J., Fanning, S., and McMahon, B. J. (2015). Antimicrobial Resistance in Wildlife: Implications for Public Health. *Zoonoses Public Health* 62, 534–542. doi:10.1111/zph.12182.

Carter, D. L., Docherty, K. M., Gill, S. A., Baker, K., Teachout, J., and Vonhof, M. J. (2018). Antibiotic resistant bacteria are widespread in songbirds across rural and urban environments. *Sci. Total Environ.* 627, 1234–1241. doi:10.1016/j.scitotenv.2018.01.343.

Casey, C. L., Hernandez, S. M., Yabsley, M. J., Smith, K. F., and Sanchez, S. (2015). The carriage of antibiotic resistance by enteric bacteria from imported tokay geckos (Gekko gecko) destined for the pet trade. *Sci. Total Environ.* 505, 299–305. doi:10.1016/j.scitotenv.2014.09.102.

Čížek, A., Dolejská, M., Karpíšková, R., Dědičová, D., Literák, I., Cizek, A., et al. (2007). Wild black-headed gulls (Larus ridibundus) as an environmental reservoir of Salmonella strains resistant to antimicrobial drugs. *Eur. J. Wildl. Res.* 53, 55–60. doi:10.1007/s10344-006-0054-2.

Claudio, V. C., Gonzalez, I., Barbosa, G., Rocha, V., Moratelli, R., and Rassy, F. (2018). Bacteria richness and antibiotic-resistance in bats from a protected area in the Atlantic Forest of Southeastern Brazil. *PLoS One* 13. doi:10.1371/journal.pone.0203411.

Cole, D., Drum, D. J. V, Stallknecht, D. E., White, D. G., Lee, M. D., Ayers, S., et al. (2005). Free-living Canada Geese and antimicrobial resistance. *Emerg. Infect. Dis.* 11, 935–938.

Costa, D., Poeta, P., Sáenz, Y., Vinué, L., Coelho, A. C., Matos, M., et al. (2008). Mechanisms of antibiotic resistance in Escherichia coli isolates recovered from wild animals. *Microb. Drug Resist.* 14, 71–77.

Costa, D., Poeta, P., Saenz, Y., Vinue, L., Rojo-Bezares, B., Jouini, A., et al. (2006). Detection of Escherichia coli harbouring extended-spectrum beta-lactamases of the CTX-M, TEM and SHV classes in faecal samples of wild animals in Portugal. *J. Antimicrob. Chemother.* 58, 1311–1312. doi:10.1093/jac/dkl415.

Cristobal-Azkarate, J., Dunn, J. C., Day, J. M. W., and Amabile-Cuevas, C. F. (2014). Resistance to Antibiotics of Clinical Relevance in the Fecal Microbiota of Mexican Wildlife. *PLoS One* 9. doi:10.1371/journal.pone.0107719.

Delport, T. C., Harcourt, R. G., Beaumont, L. J., Webster, K. N., and Power, M. L. (2015). MOLECULAR DETECTION OF ANTIBIOTIC-RESISTANCE DETERMINANTS IN ESCHERICHIA COLI ISOLATED FROM THE ENDANGERED AUSTRALIAN SEA LION (NEOPHOCA CINEREA). *J. Wildl. Dis.* 51, 555–563. doi:10.7589/2014-08-200.

Desvars-Larrive, A., Ruppitsch, W., Lepuschitz, S., Szostak, M.P., Spergser, J., Feßler, A.T., Schwarz, S., Monecke, S., Ehricht, R., Walzer, C. and Loncaric, I. (2019). Urban brown rats (Rattus norvegicus) as possible source of multidrug-resistant Enterobacteriaceae and meticillin-resistant Staphylococcus spp., Vienna, Austria, 2016 and 2017. *Eurosurveillance*, 24(32), 1900149.

Dias, C., Borges, A., Oliveira, D., Martinez-Murcia, A., Saavedra, M. J., and Simoes, M. (2018). Biofilms and antibiotic susceptibility of multidrug-resistant bacteria from wild animals. *PeerJ* 6. doi:10.7717/peerj.4974.

Dias, D., Torres, R. T., Kronvall, G., Fonseca, C., Mendo, S., and Caetano, T. (2015). Assessment of antibiotic resistance of Escherichia coli isolates and screening of Salmonella spp. in wild ungulates from Portugal. *Res. Microbiol.* 166. doi:10.1016/j.resmic.2015.03.006.

Dobbin, G., Hariharan, H., Daoust, P. Y., Hariharan, S., Heaney, S., Coles, M., et al. (2005). Bacterial flora of free-living Double-crested cormorant (Phalacrocorax auritus) chicks on Prince Edward Island, Canada, with reference to enteric bacteria and antibiotic resistance. *Comp. Immunol. Microbiol. Infect. Dis.* 28, 71–82. doi:10.1016/j.cimid.2004.08.001.

Dolejska, M., Bierosova, B., Kohoutova, L., Literak, I., and Cizek, A. (2009). Antbiotic-resistant Salmonella and Escherichia coli isolates with integrons and extended-spectrum beta-lactamases in surface water and sympatric black-headed gulls. *J. Appl. Microbiol.* 106, 1941–1950. doi:10.1111/j.1365-2672.2009.04155.x.

Dolejska, M., Cizek, A., and Literak, I. (2007). High prevalence of antimicrobial-resistant genes and integrons in Escherichia coli isolates from Black-headed Gulls in the Czech Republic. *J. Appl. Microbiol.* 103, 11–19. doi:10.1111/j.1365-2672.2006.03241.x.

Dudzic, A., Urban-Chmiel, R., Stepien-Pysniak, D., Dec, M., Puchalski, A., and Wernicki, A. (2016). Isolation, identification and antibiotic resistance of Campylobacter strains isolated from domestic and free-living pigeons. *Br. Poult. Sci.* 57, 172–178. doi:10.1080/00071668.2016.1148262.

Fallacara, D. M., Monahan, C. M., Morishita, T. Y., and Wack, R. F. (2001). Fecal shedding and antimicrobial susceptibility of selected bacterial pathogens and a survey of intestinal parasites in free-living waterfowl. *AVIAN Dis.* 45, 128–135. doi:10.2307/1593019.

Foti, M., Giacopello, C., Bottari, T., Fisichella, V., Rinaldo, D., and Mammina, C. (2009). Antibiotic Resistance of Gram Negatives isolates from loggerhead sea turtles (Caretta caretta) in the central Mediterranean Sea. *Mar. Pollut. Bull.* 58, 1363–1366. doi:10.1016/j.marpolbul.2009.04.020.

Foti, M., Mascetti, A., Fisichella, V., Fulco, E., Orlandella, B. M., and Lo Piccolo, F. (2017). Antibiotic resistance assessment in bacteria isolated in migratory Passeriformes transiting through the Metaponto territory (Basilicata, Italy). *AVIAN Res.* 8. doi:10.1186/s40657-017-0085-2.

Foti, M., Rinaldo, D., Guercio, A., Giacopello, C., Aleo, A., De Leo, F., et al. (2011). Pathogenic microorganisms carried by migratory birds passing through the territory of the island of Ustica, Sicily (Italy). *AVIAN Pathol.* 40, 405–409. doi:10.1080/03079457.2011.588940.

Foti, M., Siclari, A., Mascetti, A., and Fisichella, V. (2018). Study of the spread of antimicrobial-resistant Enterobacteriaceae from wild mammals in the National Park of Aspromonte (Calabria, Italy). *Environ. Toxicol. Pharmacol.* 63, 69–73. doi:10.1016/j.etap.2018.08.016.

Fresno, M., Barrera, V., Gornall, V., Lillo, P., Paredes, N., Abalos, P., et al. (2013). Identification of diverse Salmonella Serotypes, Virulotypes, and Antimicrobial Resistance Phenotypes in Waterfowl From Chile. *VECTOR-BORNE ZOONOTIC Dis.* 13, 884–887. doi:10.1089/vbz.2013.1408.

Furness, L. E., Campbell, A., Zhang, L., Gaze, W. H., and McDonald, R. A. (2017). Wild small mammals as sentinels for the environmental transmission of antimicrobial resistance. *Environ. Res.* 154, 28–34. doi:10.1016/j.envres.2016.12.014.

Gaukler, S. M., Linz, G. M., Sherwood, J. S., Dyer, N. W., Bleier, W. J., Wannemuehler, Y. M., Nolan, L. K., et al. (2009). Escherichia coli, Salmonella, and Mycobacterium avium subsp. paratuberculosis in wild European starlings at a Kansas cattle feedlot. *Avian Dis.* 53, 544–551.

Ghanbarpour, R., and Daneshdoost, S. (2012). Identification of shiga toxin and intimin coding genes in Escherichia coli isolates from pigeons (Columba livia) in relation to phylotypes and antibiotic resistance patterns. *Trop. Anim. Health Prod.* 44, 307–312. doi:10.1007/s11250-011-0021-0.

Giacopello, C., Foti, M., Mascetti, A., Grosso, F., Ricciardi, D., Fisichella, V., et al. (2016). Antimicrobial resistance patterns of Enterobacteriaceae in European wild bird species admitted in a wildlife rescue centre. *Vet. Ital.* 52, 139–144. doi:10.12834/VetIt.327.1374.2.

Gibbs, P. S., Kasa, R., Newbrey, J. L., Petermann, S. R., Wooley, R. E., Vinson, H. M., et al. (2007). Identification, antimicrobial resistance profiles, and virulence of members from the family Enterobacteriaceae from the feces of yellow-headed blackbirds (Xanthocephalus xanthocephalus) in North Dakota. *Avian Dis.* 51, 649–55. doi:10.1637/0005-2086(2007)51[649:Iarpav]2.0.Co;2.

Gilliver, M. A., Bennett, M., Begon, M., Hazel, S. M., and Hart, C. A. (1999). Enterobacteria: antibiotic resistance found in wild rodents. *Nature* 401, 233–234. doi:10.1038/35051176.

Gionechetti, F., Zucca, P., Gombac, F., Monti-Bragadin, C., Lagatolla, C., Tonin, E., et al. (2008). Characterization of antimicrobial resistance and class 1 integrons in Enterobacteriaceae isolated from Mediterranean herring gulls (Larus cachinnans). *Microb. DRUG Resist.* 14, 93–99. doi:10.1089/mdr.2008.0803.

Gómez, P., Lozano, C., Camacho, M.C., Lima-Barbero, J.F., Hernández, J.M., Zarazaga, M., Höfle, Ú. and Torres, C., 2016. Detection of MRSA ST3061-t843-mecC and ST398-t011-mecA in white stork nestlings exposed to human residues. *J. Antimic. Chemo*, *71*(1), pp.53-57.

Goncalves, A., Igrejas, G., Radhouani, H., Correia, S., Pacheco, R., Santos, T., et al. (2013). Antimicrobial resistance in faecal enterococci and Escherichia coli isolates recovered from Iberian wolf. *Lett. Appl. Microbiol.* 56, 268–274. doi:10.1111/lam.12044.

Goncalves, A., Igrejas, G., Radhouani, H., Santos, T., Monteiro, R., Pacheco, R., et al. (2013). Detection of antibiotic resistant enterococci and Escherichia coli in free range Iberian Lynx (Lynx pardinus). *Sci. Total Environ.* 456, 115–119. doi:10.1016/j.scitotenv.2013.03.073.

Gorski, L., Jay-Russell, M. T., Liang, A. S., Walker, S., Bengson, Y., Govoni, J., et al. (2013). Diversity of pulsed-field gel electrophoresis pulsotypes, serovars, and antibiotic resistance among Salmonella isolates from wild amphibians and reptiles in the California Central Coast. *Foodborne Pathog. Dis.* 10, 540–548. doi:10.1089/fpd.2012.1372.

Grall, N., Barraud, O., Wieder, I., Hua, A., Perrier, M., Babosan, A., et al. (2015). Lack of dissemination of acquired resistance to -lactams in small wild mammals around an isolated village in the Amazonian forest. *Environ. Microbiol. Rep.* 7, 698–708. doi:10.1111/1758-2229.12289.

Graves, A. K., Hagedorn, C., Teetor, A., Mahal, M., Booth, A. M., and Reneau, R. B. (2002). Antibiotic resistance profiles to determine sources of fecal contamination in a rural Virginia watershed. *J. Environ. Qual.* 31, 1300–1308.

GRAVES, S. R., KENNELLYMERRIT, S. A., TIDEMANN, C. R., RAWLINSON, P. A., HARVEY, K. J., and THORNTON, I. W. B. (1988). ANTIBIOTIC-RESISTANCE PATTERNS OF ENTERIC BACTERIA OF WILD MAMMALS ON THE KRAKATAU ISLANDS AND WEST JAVA, INDONESIA. *Philos. Trans. R. Soc. LONDON Ser. B-BIOLOGICAL Sci.* 322, 339–353. doi:10.1098/rstb.1988.0129.

Greig, J., Rajić, A., Young, I., Mascarenhas, M., Waddell, L., and Lejeune, J. (2015). A scoping review of the role of wildlife in the transmission of bacterial pathogens and antimicrobial resistance to the food chain. *Zoonoses Public Health* 62, 269–284. doi:10.1111/zph.12147.

Guenther, S., Grobbel, M., Heidemanns, K., Schlegel, M., Ulrich, R. G., Ewers, C., et al. (2010). First insights into antimicrobial resistance among faecal Escherichia coli isolates from small wild mammals in rural areas. *Sci. Total Environ.* 408, 3519–3522. doi:10.1016/j.scitotenv.2010.05.005.

Guenther, S., Grobbel, M., Luebke-Becker, A., Goedecke, A., Friedrich, N. D., Wieler, L. H., et al. (2010). Antimicrobial resistance profiles of Escherichia coli from common European wild bird species. *Vet. Microbiol.* 144, 219–225. doi:10.1016/j.vetmic.2009.12.016.

Guerra, B., Fischer, J., and Helmuth, R. (2014). An emerging public health problem: Acquired carbapenemase-producing microorganisms are present in food-producing animals, their environment, companion animals and wild birds. *Vet. Microbiol.* 171, 290–297. doi:10.1016/j.vetmic.2014.02.001.

Hacioglu, N., and Tosunoglu, M. (2014). Determination of antimicrobial and heavy metal resistance profiles of some bacteria isolated from aquatic amphibian and reptile species. *Environ. Monit. Assess.* 186, 407–413. doi:10.1007/s10661-013-3385-y

Hagedorn, C., Robinson, S. L., Filtz, J. R., Grubbs, S. M., Angier, T. A., and Reneau, R. B. (1999). Determining sources of fecal pollution in a rural virginia watershed with antibiotic resistance patterns in fecal streptococci. *Appl. Environ. Microbiol.* 65, 5522–5531.

Hamarova, L., Repel, M., Javorsky, P., and Pristas, P. (2017). Evaluation of enteromicroflora of common crane (Grus grus) as a potential reservoir of bacterial antimicrobial resistance. *Biologia (Bratisl).* 72, 1098–1100. doi:10.1515/biolog-2017-0118.

Handeland, K., Refsum, T., Johansen, B. S., Holstad, G., Knutsen, G., Solberg, I., et al. (2002). Prevalence of Salmonella typhimurium infection in Norwegian hedgehog populations associated with two human disease outbreaks. *Epidemiol. Infect.* 128, 523–527. doi:10.1017/S0950268802007021.

Handrova, L., and Kmet, V. (2019). Antibiotic resistance and virulence factors of Escherichia coli from eagles and goshawks. *J. Environ. Sci. Heal. PART B-PESTICIDES FOOD Contam. Agric. WASTES* 54, 605–614. doi:10.1080/03601234.2019.1608103.

Hasan, B., Laurell, K., Rakib, M. M., Ahlstedt, E., Hernandez, J., Caceres, M., et al. (2016). Fecal Carriage of Extended-Spectrum -Lactamases in Healthy Humans, Poultry, and Wild Birds in Leon, NicaraguaA Shared Pool of bla(CTX-M) Genes and Possible Interspecies Clonal Spread of Extended-Spectrum -Lactamases-Producing Escherichia coli. *Microb. DRUG Resist.* 22, 682–687. doi:10.1089/mdr.2015.0323.

Hasan, B., Melhus, A., Sandegren, L., Alam, M., and Olsen, B. (2014). The Gull (Chroicocephalus brunnicephalus) as an Environmental Bioindicator and Reservoir for Antibiotic Resistance on the Coastlines of the Bay of Bengal. *Microb. DRUG Resist.* 20, 466–471. doi:10.1089/mdr.2013.0233.

Hasan, B., Sandegren, L., Melhus, Å., Drobni, M., Hernandez, J., Waldenström, J., et al. (2012). Antimicrobial drug-resistant escherichia coli in wild birds and free-range poultry, Bangladesh. *Emerg. Infect. Dis.* 18, 2055–2058. doi:10.3201/eid1812.120513.

Hernandez, J., Johansson, A., Stedt, J., Bengtsson, S., Porczak, A., Granholm, S., et al. (2013). Characterization and Comparison of Extended-Spectrum beta-Lactamase (ESBL) Resistance Genotypes and Population Structure of Escherichia coli Isolated from Franklin’s Gulls (Leucophaeus pipixcan) and Humans in Chile. *PLoS One* 8. doi:10.1371/journal.pone.0076150.

Hessman, J., Atterby, C., Olsen, B., and Jarhult, J. D. (2018). High Prevalence and Temporal Variation of Extended Spectrum -Lactamase-Producing Bacteria in Urban Swedish Mallards. *Microb. DRUG Resist.* 24, 822–829. doi:10.1089/mdr.2017.0263.

Iglesias-Torrens, Y., Miro, E., Guirado, P., Llovet, T., Munoz, C., Cerda-Cuellar, M., et al. (2018). Population Structure, Antimicrobial Resistance, and Virulence-Associated Genes in Campylobacter jejuni Isolated From Three Ecological Niches: Gastroenteritis Patients, Broilers, and Wild Birds. *Front. Microbiol.* 9. doi:10.3389/fmicb.2018.01676.

Ishibashi, S., Sumiyama, D., Kanazawa, T., and Murata, K. (2019). Prevalence of antimicrobial-resistant Escherichia coli in endangered Okinawa rail (Gallirallus okinawae) inhabiting areas around a livestock farm. *Vet. Med. Sci.* doi:10.1002/vms3.194.

Janatova, M., Albrechtova, K., Petrzelkova, K. J., Dolejska, M., Papousek, I., Masarikova, M., et al. (2014). Antimicrobial-resistant Enterobacteriaceae from humans and wildlife in Dzanga-Sangha Protected Area, Central African Republic. *Vet. Microbiol.* 171, 422–431. doi:10.1016/j.vetmic.2014.02.014.

Janecko, N., Cizek, A., Halova, D., Karpiskova, R., Myskova, P., and Literak, I. (2015). Prevalence, Characterization and Antibiotic Resistance of Salmonella Isolates in Large Corvid Species of Europe and North America Between 2010 and 2013. *Zoonoses Public Health* 62, 292–300. doi:10.1111/zph.12149.

Jardine, C. M., Janecko, N., Allan, M., Boerlin, P., Chalmers, G., Kozak, G., et al. (2012). Antimicrobial Resistance in Escherichia coli Isolates from Raccoons (Procyon lotor) in Southern Ontario, Canada. *Appl. Environ. Microbiol.* 78, 3873–3879. doi:10.1128/AEM.00705-12.

Jobbins, S. E., and Alexander, K. A. (2015). FROM WHENCE THEY CAME-ANTIBIOTIC-RESISTANT ESCHERICHIA COLI IN AFRICAN WILDLIFE. *J. Wildl. Dis.* 51, 811–820. doi:10.7589/2014-11-257.

Jurado-Tarifa, E., Torralbo, A., Borge, C., Cerda-Cuellar, M., Ayats, T., Carbonero, A., et al. (2016). Genetic diversity and antimicrobial resistance of Campylobacter and Salmonella strains isolated from decoys and raptors. *Comp. Immunol. Microbiol. Infect. Dis.* 48, 14–21. doi:10.1016/j.cimid.2016.07.003.

Klibi, N., Ben Amor, I., Rahmouni, M., Dziri, R., Douja, G., Ben Said, L., et al. (2015). Diversity of species and antibiotic resistance among fecal enterococci from wild birds in Tunisia. Detection of vanA-containing Enterococcus faecium isolates. *Eur. J. Wildl. Res.* 61, 319–323. doi:10.1007/s10344-014-0884-2.

Kmet, V., Cuvalova, A., and Stanko, M. (2018). Small mammals as sentinels of antimicrobial-resistant staphylococci. *Folia Microbiol. (Praha).* 63, 665–668. doi:10.1007/s12223-018-0594-3.

Kozak, G. K., Boerlin, P., Janecko, N., Reid-Smith, R. J., and Jardine, C. (2009). Antimicrobial Resistance in Escherichia coli Isolates from Swine and Wild Small Mammals in the Proximity of Swine Farms and in Natural Environments in Ontario, Canada. *Appl. Environ. Microbiol.* 75, 559–566. doi:10.1128/AEM.01821-08.

Lanthier, M., Scott, A., Lapen, D. R., Zhang, Y., and Topp, E. (2010). Frequency of virulence genes and antibiotic resistances in Enterococcus spp. isolates from wastewater and feces of domesticated mammals and birds, and wildlife. *Can. J. Microbiol.* 56, 715–729. doi:10.1139/W10-046.

Leatherbarrow, A. J. H., Griffiths, R., Hart, C. A., Kemp, R., Williams, N. J., Diggle, P. J., et al. (2007). Campylobacter lari: Genotype and antibiotic resistance of isolates from cattle, wildlife and water in an area of mixed dairy farmland in the United Kingdom. *Environ. Microbiol.* 9, 1772–1779. doi:10.1111/j.1462-2920.2007.01295.x.

Lessa, S. S., Paes, R. C. S., Santoro, P. N., Mauro, R. A., and Vieira-da-Motta, O. (2011). Identification and Antimicrobial Resistance of Microflora Colonizing Feral Pig (Sus Scrofa) of Brazilian Pantanal. *Brazilian J. Microbiol.* 42, 740–749.

Lillehaug, A., Bergsjo, B., Schau, J., Bruheim, T., Vikoren, T., and Handeland, K. (2005). Campylobacter spp., Salmonella spp., verocytotoxic Escherichia coli, and antibiotic resistance in indicator organisms in wild cervids. *ACTA Vet. Scand.* 46, 23–32. doi:10.1186/1751-0147-46-23.

Literak, I., Dolejska, M., Radimersky, T., Klimes, J., Friedman, M., Aarestrup, F. M., et al. (2010). Antimicrobial-resistant faecal Escherichia coli in wild mammals in central Europe: multiresistant Escherichia coli producing extended-spectrum beta-lactamases in wild boars. *J. Appl. Microbiol.* 108, 1702–1711. doi:10.1111/j.1365-2672.2009.04572.x.

Literak, I., Vanko, R., Dolejska, M., Cizek, A., and Karpiskova, R. (2007). Antibiotic resistant Escherichia coli and Salmonella in Russian rooks (Corvus frugilegus) wintering in the Czech Republic. *Lett. Appl. Microbiol.* 45, 616–621. doi:10.1111/j.1472-765X.2007.02236.x.

Literak, I., Dolejska, M., Cizek, A., Djigo, C. A. T., Konecny, A., and Koubek, P. (2009). Reservoirs of antibiotic-resistant Enterobacteriaceae among animals sympatric to humans in Senegal: Extended-spectrum beta-lactamases in bacteria in a black rat (Rattus rattus). *African J. Microbiol. Res.* 3, 751–754. Available at: http://www.academicjournals.org/journal/AJMR/article-abstract/7F1491E14717.

Literak, I., Dolejska, M., Janoszowska, D., Hrusakova, J., Meissner, W., Rzyska, H., et al. (2010). Antibiotic-Resistant Escherichia coli Bacteria, Including Strains with Genes Encoding the Extended-Spectrum Beta-Lactamase and QnrS, in Waterbirds on the Baltic Sea Coast of Poland. *Appl. Environ. Microbiol.* 76, 8126–8134. doi:10.1128/AEM.01446-10.

Liu, D., Wilson, C., Hearlson, J., Singleton, J., Thomas, R. B., and Crupper, S. S. (2013). PREVALENCE OF ANTIBIOTIC-RESISTANT GRAM-NEGATIVE BACTERIA ASSOCIATED WITH THE RED-EARED SLIDER (TRACHEMYS SCRIPTA ELEGANS). *J. ZOO Wildl. Med.* 44, 666–671. doi:10.1638/2012-0252R1.1.

Livermore, D. M., Warner, M., Hall, L. M. C., Enne, V. I., Projan, S. J., Dunman, P. M., et al. (2001). Antibiotic resistance in bacteria from magpies (Pica pica) and rabbits (Oryctolagus cuniculus) from west Wales. *Environ. Microbiol.* 3, 658–661. doi:10.1046/j.1462-2920.2001.00239.x.

Loncaric, I., Stalder, G.L., Mehinagic, K., Rosengarten, R., Hoelzl, F., Knauer, F. and Walzer, C. (2013). Comparison of ESBL–and AmpC producing Enterobacteriaceae and methicillin-resistant Staphylococcus aureus (MRSA) isolated from migratory and resident population of rooks (Corvus frugilegus) in Austria. *PLoS One*, 8(12), p.e84048.

Lozano, C., Gonzalez-Barrio, D., Garcia, J. T., Ceballos, S., Olea, P. P., Ruiz-Fons, F., et al. (2015). Detection of vancomycin-resistant Enterococcus faecalis ST6-vanB2 and E. faecium ST915-vanA in faecal samples of wild Rattus rattus in Spain. *Vet. Microbiol.* 177, 168–174. doi:10.1016/j.vetmic.2015.02.025.

Luzzago, C., Locatelli, C., Franco, A., Scaccabarozzi, L., Gualdi, V., Vigano, R., et al. (2014). Clonal diversity, virulence-associated genes and antimicrobial resistance profile of Staphylococcus aureus isolates from nasal cavities and soft tissue infections in wild ruminants in Italian Alps. *Vet. Microbiol.* 170, 157–161. doi:10.1016/j.vetmic.2014.01.016.

MacDonald, A. M., Jardine, C. M., Susta, E., Slavic, D., and Nemeth, N. M. (2018). Survey for Bacteria and Antimicrobial Resistance in Wild Turkeys (Meleagris gallopavo) in Ontario, Canada. *AVIAN Dis.* 62, 184–188. doi:10.1637/11807-020618-Reg.1.

Machado, D. N., Lopes, E. S., Albuquerque, A. H., Horn V, R., Bezerra, W. G. A., Siqueira, R. A. S., et al. (2018). Isolation and Antimicrobial Resistance Profiles of Enterobacteria from Nestling Grey-Breasted Parakeets (Pyrrhura Griseipectus). *BRAZILIAN J. Poult. Sci.* 20, 103–110. doi:10.1590/1806-9061-2017-0551.

Mallon, D. J., Corkill, J. E., Hazel, S. M., Wilson, J. S., French, N. P., Bennett, M., et al. (2002). Excretion of vancomycin-resistant enterococci by wild mammals. *Emerg. Infect. Dis.* 8, 636–638.

Marcelino, V. R., Wille, M., Hurt, A. C., Gonzalez-Acuna, D., Klaassen, M., Schlub, T. E., et al. (2019). Meta-transcriptomics reveals a diverse antibiotic resistance gene pool in avian microbiomes. *BMC Biol.* 17. doi:10.1186/s12915-019-0649-1.

Marinho, C., Igrejas, G., Goncalves, A., Silva, N., Santos, T., Monteiro, R., et al. (2014). Azorean wild rabbits as reservoirs of antimicrobial resistant Escherichia coli. *Anaerobe* 30, 116–119. doi:10.1016/j.anaerobe.2014.09.009.

Marinho, C., Silva, N., Pombo, S., Santos, T., Monteiro, R., Goncalves, A., et al. (2013). Echinoderms from Azores islands: An unexpected source of antibiotic resistant Enterococcus spp. and Escherichia coli isolates. *Mar. Pollut. Bull.* 69, 122–127. doi:10.1016/j.marpolbul.2013.01.017.

Martiny, A. C., Martiny, J. B. H., Weihe, C., Field, A., and Ellis, J. C. (2011). Functional metagenomics reveals previously unrecognized diversity of antibiotic resistance genes in gulls. *Front. Microbiol.* 2. doi:10.3389/fmicb.2011.00238.

McDougall, F., Boardman, W., Gillings, M., and Power, M. (2019). Bats as reservoirs of antibiotic resistance determinants: A survey of class 1 integrons in Grey-headed Flying Foxes (Pteropus poliocephalus). *Infect. Genet. Evol.* 70, 107–113. doi:10.1016/j.meegid.2019.02.022.

Merkeviciene, L., Ruzauskaite, N., Klimiene, I., Siugzdiniene, R., Dailidaviciene, J., Virgailis, M., et al. (2017). Microbiome and antimicrobial resistance genes in microbiota of cloacal samples from European herring gulls (Larus argentatus). *J. Vet. Res.* 61, 27–35. doi:10.1515/jvetres-2017-0004.

Middleton, J. H., and Ambrose, A. (2005). Enumeration and antibiotic resistance patterns of fecal indicator organisms isolated from migratory Canada geese (Branta canadensis). *J. Wildl. Dis.* 41, 334–341.

Migura-Garcia, L., Ramos, R., and Cerda-Cuellar, M. (2017). Antimicrobial Resistance of Salmonella Serovars and Campylobacter spp. Isolated from an Opportunistic Gull Species, Yellow-legged Gull (Larus michahellis). *J. Wildl. Dis.* 53, 148–152. doi:10.7589/2016-03-051.

Miller, E. A., Johnson, T. J., Omondi, G., Atwill, E. R., Isbell, L. A., McCowan, B., et al. (2019). Assessing Transmission of Antimicrobial-Resistant Escherichia coli in Wild Giraffe Contact Networks. *Appl. Environ. Microbiol.* 85. doi:10.1128/AEM.02136-18.

Miller, R. V., Gammon, K., and Day, M. J. (2009). Antibiotic resistance among bacteria isolated from seawater and penguin fecal samples collected near Palmer Station, Antarctica. *Can. J. Microbiol.* 55, 37–45. doi:10.1139/W08-119.

Mo, S. S., Urdahl, A. M., Madslien, K., Sunde, M., Nesse, L. L., Slettemeas, J. S., et al. (2018). What does the fox say? Monitoring antimicrobial resistance in the environment using wild red foxes as an indicator. *PLoS One* 13. doi:10.1371/journal.pone.0198019.

Molina-Lopez, R. A., Valveru, N., Martin, M., Mateu, E., Obon, E., Cerda-Cuellar, M., et al. (2011). Wild raptors as carriers of antimicrobial-resistant Salmonella and Campylobacter strains. *Vet. Rec.* 168. doi:10.1136/vr.c7123.

Monecke, S., Gavier-Widen, D., Mattsson, R., Rangstrup-Christensen, L., Lazaris, A., Coleman, D. C., et al. (2013). Detection of mecC-Positive Staphylococcus aureus (CC130-MRSA-XI) in Diseased European Hedgehogs (Erinaceus europaeus) in Sweden. *PLoS One* 8, 4–9. doi:10.1371/journal.pone.0066166.

More, E., Ayats, T., Ryan, P. G., Naicker, P. R., Keddy, K. H., Gaglio, D., et al. (2017). Seabirds (Laridae) as a source of Campylobacter spp., Salmonella spp. and antimicrobial resistance in South Africa. *Environ. Microbiol.* 19, 4164–4176. doi:10.1111/1462-2920.13874.

Mukerji, S., Stegger, M., Truswell, A. V., Laird, T., Jordan, D., Abraham, R. J., et al. (2019). Resistance to critically important antimicrobials in Australian silver gulls (Chroicocephalus novaehollandiae) and evidence of anthropogenic origins. *J. Antimicrob. Chemother.* 74, 2566–2574. doi:10.1093/jac/dkz242.

Murugaiyan, J., Krueger, K., Roesler, U., Weinreich, J., and Schierack, P. (2015). Assessment of species and antimicrobial resistance among Enterobacteriaceae isolated from mallard duck faeces. *Environ. Monit. Assess.* 187. doi:10.1007/s10661-015-4346-4.

Nakamura, I., Obi, T., Sakemi, Y., Nakayama, A., Miyazaki, K., Ogura, G., et al. (2011). The Prevalence of Antimicrobial-Resistant Escherichia coli in Two Species of Invasive Alien Mammals in Japan. *J. Vet. Med. Sci.* 73, 1067–1070.

Nascimento, A. M. A., Cursino, L., Goncalves-Dornelas, H., Reis, A., Chartone-Souza, E., and Marini, M. A. (2003). Antibiotic-resistant gram-negative bacteria in birds from the Brazilian Atlantic Forest. *Condor* 105, 358–361. doi:10.1650/0010-5422(2003)105{[}0358:AGBIBF]2.0.CO;2.

Navarro-Gonzalez, N., Porrero, M. C., Mentaberre, G., Serrano, E., Mateos, A., Domínguez, L., et al. (2013). Antimicrobial resistance in indicator escherichia coli isolates from free-ranging livestock and sympatric wild ungulates in a natural environment (Northeastern Spain). *Appl. Environ. Microbiol.* 79, 6184–6186. doi:10.1128/AEM.01745-13.

Navarro-Gonzalez, N., Casas-Diaz, E., Porrero, C. M., Mateos, A., Dominguez, L., Lavin, S., et al. (2013). Food-borne zoonotic pathogens and antimicrobial resistance of indicator bacteria in urban wild boars in Barcelona, Spain. *Vet. Microbiol.* 167, 686–689. doi:10.1016/j.vetmic.2013.07.037.

Navarro-Gonzalez, N., Castillo-Contreras, R., Casas-Diaz, E., Morellet, N., Concepcion Porrero, M., Molina-Vacas, G., et al. (2018). Carriage of antibiotic-resistant bacteria in urban versus rural wild boars. *Eur. J. Wildl. Res.* 64. doi:10.1007/s10344-018-1221-y.

Navarro-Gonzalez, N., Mentaberre, G., Porrero, C. M. C. M. C. M., Serrano, E., Mateos, A., Lopez-Martin, J. M., et al. (2012). Effect of Cattle on Salmonella Carriage, Diversity and Antimicrobial Resistance in Free-Ranging Wild Boar (Sus scrofa) in Northeastern Spain. *PLoS One* 7, e51614. doi:10.1371/journal.pone.0051614.

Nhung, N. T., Cuong, N. V., Campbell, J., Hoa, N. T., Bryant, J. E., Truc, V. N. T., et al. (2015). High levels of antimicrobial resistance among Escherichia Coli isolates from livestock farms and synanthropic rats and shrews in the mekong delta of Vietnam. *Appl. Environ. Microbiol.* 81, 812–820. doi:10.1128/AEM.03366-14.

Nieto-Claudin, A., Esperon, F., Blake, S., and Deem, S. L. (2019). Antimicrobial resistance genes present in the faecal microbiota of free-living Galapagos tortoises (Chelonoidis porteri). *Zoonoses Public Health* 66, 900–908. doi:10.1111/zph.12639.

Ogawa, K., Yamaguchi, K., Suzuki, M., Tsubota, T., Ohya, K., and Fukushi, H. (2011). GENETIC CHARACTERISTICS AND ANTIMICROBIAL RESISTANCE OF ESCHERICHIA COLI FROM JAPANESE MACAQUES (MACACA FUSCATA) IN RURAL JAPAN. *J. Wildl. Dis.* 47, 261–270.

Oliveira, M., Pedroso, N. M., Sales-Luis, T., Santos-Reis, M., Tavares, L., and Vilela, C. L. (2010). Antimicrobial-Resistant Salmonella Isolated from Eurasian Otters (Lutra lutra Linnaeus, 1758) in Portugal. *J. Wildl. Dis.* 46, 1257–1261.

Oluduro, A. O. (2012). Antibiotic-resistant commensal Escherichia coli in faecal droplets from bats and poultry in Nigeria. *Vet. Ital.* 48, 297–308. Available at: http://www.ncbi.nlm.nih.gov/pubmed/23038076.

Osterblad, M., Norrdahl, K., Korpimaki, E., and Huovinen, P. (2001). Antibiotic resistance - How wild are wild mammals? *Nature* 409, 37–38. doi:10.1038/35051173.

Palmgren, H., Sellin, M., Bergström, S., and Olsen, B. (1997). Enteropathogenic bacteria in migrating birds arriving in Sweden. *Scand. J. Infect. Dis.* 29, 565–568.

Palomo, G., Campos, M. J., Ugarte, M., Porrero, M. C., Alonso, J. M., Borge, C., et al. (2013). Dissemination of antimicrobial-resistant clones of Salmonella enterica among domestic animals, wild animals, and humans. *Foodborne Pathog. Dis.* 10, 171–6. doi:10.1089/fpd.2012.1288.

Patel, A., Lloyd, D. H., and Lamport, A. I. (1999). Antimicrobial resistance of feline staphylococci in southeastern England. *Vet. Dermatol.* 10, 257–261. doi:10.1046/j.1365-3164.1999.00178.x.

Pavlickova, S., Klancnik, A., Dolezalova, M., Mozina, S. S., and Holko, I. (2017). Antibiotic resistance, virulence factors and biofilm formation ability in Escherichia coli strains isolated from chicken meat and wildlife in the Czech Republic. *J. Environ. Sci. Heal. PART B-PESTICIDES FOOD Contam. Agric. WASTES* 52, 570–576. doi:10.1080/03601234.2017.1318637.

Poeta, P., Costa, D., Igrejas, I., Rojo-Bezares, B., Sáenz, Y., Zarazaga, M., et al. (2007). Characterization of vanA-Containing Enterococcus faecium Isolates Carrying Tn5397-Like and Tn916/Tn1545-Like Transposons in Wild Boars (Sus Scrofa). *Microb. Drug Resist.* 13, 151–156.

Poeta, P., Costa, D., Rodrigues, J., and Torres, C. (2005). Study of faecal colonization by vanA-containing Enterococcus strains in healthy humans, pets, poultry and wild animals in Portugal. *J. Antimicrob. Chemother. 55(2), 278-280.* 55, 278–280.

Poeta, P., Costa, D., Sáenz, Y., Klibi, N., Ruiz‐Larrea, F., Rodrigues, J., et al. (2005). Characterization of antibiotic resistance genes and virulence factors in faecal enterococci of wild animals in Portugal. *J. Vet. Med. Ser. B* 52, 396–402.

Poeta, P., Costa, D., Igrejas, G., Rodrigues, J., and Torres, C. (2007). Phenotypic and genotypic characterization of antimicrobial resistance in faecal enterococci from wild boars (Sus scrofa). *Vet. Microbiol.* 125, 368–74. doi:10.1016/j.vetmic.2007.06.003.

Porrero, M.C., Mentaberre, G., Sánchez, S., Fernández-Llario, P., Gómez-Barrero, S., Navarro-Gonzalez, N., Serrano, E., Casas-Díaz, E., Marco, I., Fernández-Garayzabal, J.F. and Mateos, A. (2013). Methicillin resistant Staphylococcus aureus (MRSA) carriage in different free-living wild animal species in Spain. *The Vet. J.* 198(1), 127-130.

Prichula, J., Pereira, R. I., Wachholz, G. R., Cardoso, L. A., Correa Tolfo, N. C., Santestevan, N. A., et al. (2016). Resistance to antimicrobial agents among enterococci isolated from fecal samples of wild marine species in the southern coast of Brazil. *Mar. Pollut. Bull.* 105, 51–57. doi:10.1016/j.marpolbul.2016.02.071.

Rabbia, V., Bello-Toledo, H., Jimenez, S., Quezada, M., Dominguez, M., Vergara, L., et al. (2016). Antibiotic resistance in Escherichia coli strains isolated from Antarctic bird feces, water from inside a wastewater treatment plant, and seawater samples collected in the Antarctic Treaty area. *POLAR Sci.* 10, 123–131. doi:10.1016/j.polar.2016.04.002.

Radhouani, H., Igrejas, G., Carvalho, C., Pinto, L., Gonçalves, A., Lopez, M., et al. (2011). Clonal lineages, antibiotic resistance and virulence factors in vancomycin-resistant enterococci isolated from fecal samples of red foxes (vulpes vulpes). *J. Wildl. Dis.* 47, 769–773. doi:47/3/769 [pii].

Radhouani, H., Poeta, P., Igrejas, G., Gonçalves, A., Vinue, L., and Torres, C. (2009). Antimicrobial resistance and phylogenetic groups in isolates of Escherichia coli from seagulls at the Berlengas nature reserve. *Vet. Rec.* 165, 138.

Radhouani, H., Igrejas, G., Goncalves, A., Pacheco, R., Monteiro, R., Sargo, R., et al. (2013). Antimicrobial resistance and virulence genes in Escherichia coli and enterococci from red foxes (Vulpes vulpes). *Anaerobe* 23, 82–86. doi:10.1016/j.anaerobe.2013.06.013.

Radhouani, H., Poeta, P. P., Gonçalves, A., Pacheco, R., Sargo, R., Igrejas, G., et al. (2012). Wild birds as biological indicators of environmental pollution: antimicrobial resistance patterns of Escherichia coli and enterococci isolated from common buzzards (Buteo buteo). *J. Med. Microbiol.* 61, 837–843. doi:10.1099/jmm.0.038364-0.

Radhouani, H., Poeta, P., Pinto, L., Miranda, J., Coelho, C., Carvalho, C., et al. (2010). Proteomic characterization of vanA-containing Enterococcus recovered from Seagulls at the Berlengas Natural Reserve, W Portugal. *PROTEOME Sci.* 8. doi:10.1186/1477-5956-8-48.

Radimersky, T., Frolkova, P., Janoszowska, D., Dolejska, M., Svec, P., Roubalova, E., et al. (2010). Antibiotic resistance in faecal bacteria (Escherichia coli, Enterococcus spp.) in feral pigeons. *J. Appl. Microbiol.* 109, 1687–1695. doi:10.1111/j.1365-2672.2010.04797.x.

Ramey, A. M., Hernandez, J., Tyrlov, V., Uher-Koch, B. D., Schmutz, J. A., Atterby, C., et al. (2018). Antibiotic-Resistant Escherichia coli in Migratory Birds Inhabiting Remote Alaska. *Ecohealth* 15, 72–81. doi:10.1007/s10393-017-1302-5.

Ramlachan, N., Anderson, R. C., Andrews, K., Laban, G., and Nisbet, D. J. (2007). Characterization of an antibiotic resistant Clostridium hathewayi strain from a continuous-flow exclusion chemostat culture derived from the cecal contents of a feral pig. *Anaerobe* 13, 153–160. doi:10.1016/j.anaerobe.2007.03.003.

Reche, M. P., de los Ríos, J. E. G., Jiménez, P. A., Rojas, A. M., and Rotger, R. (2002). gyrA mutations associated with nalidixic acid-resistant salmonellae from wild birds. *Antimicrob. Agents Chemother.* 46, 3108–3109.

Rehman, M. U., Zhang, H., Iqbal, M. K., Nabi, F., Huang, S., Lan, Y., et al. (2017). Antibiotic Resistance of Escherichia coli in Free-Ranging Yaks (Bos grunniens) from Tibetan Plateau, China. *Pak. Vet. J.* 37, 139–144.

Rodriguez, F. I., Osinalde, J. M., Gomez, S. C., Pulido, D. G., Caffer, M. I., Nicolau, F. C., et al. (2018). Prevalence, antimicrobial resistance profile and comparison of selective plating media for the isolation of Salmonella spp. in free-ranging waterfowl from Entre Rios, Argentina. *Poult. Sci.* 97, 3043–3049. doi:10.3382/ps/pey164.

Rose, J. M., Gast, R. J., Bogomolni, A., Ellis, J. C., Lentell, B. J., Touhey, K., et al. (2009). Occurrence and patterns of antibiotic resistance in vertebrates off the Northeastern United States coast. *FEMS Microbiol. Ecol.* 67, 421–431. doi:10.1111/j.1574-6941.2009.00648.x.

ROUTMAN, E., MILLER, R. D., PHILLIPSCONROY, J., and HARTL, D. L. (1985). ANTIBIOTIC-RESISTANCE AND POPULATION-STRUCTURE IN ESCHERICHIA-COLI FROM FREE-RANGING AFRICAN YELLOW BABOONS. *Appl. Environ. Microbiol.* 50, 749–754.

Rwego, I. B., Isabirye-Basuta, G., Gillespie, T. R., and Goldberg, T. L. (2008). Gastrointestinal Bacterial Transmission among Humans, Mountain Gorillas, and Livestock in Bwindi Impenetrable National Park, Uganda. *Conserv. Biol.* 22, 1600–1607. doi:10.1111/j.1523-1739.2008.01018.x.

Sacristan, C., Esperon, F., Herrera-Leon, S., Iglesias, I., Neves, E., Nogal, V., et al. (2014). Virulence genes, antibiotic resistance and integrons in Escherichia coli strains isolated from synanthropic birds from Spain. *AVIAN Pathol.* 43, 172–175. doi:10.1080/03079457.2014.897683.

Sandegren, L., Stedt, J., Lustig, U., Bonnedahl, J., Andersson, D. I., and Jarhult, J. D. (2018). Long-term carriage and rapid transmission of extended spectrum beta-lactamase-producing E-coli within a flock of Mallards in the absence of antibiotic selection. *Environ. Microbiol. Rep.* 10, 576–582. doi:10.1111/1758-2229.12681.

Santos, T., Silva, N., Igrejas, G., Rodrigues, P., Micael, J., Rodrigues, T., et al. (2013). Dissemination of antibiotic resistant Enterococcus spp. and Escherichia coli from wild birds of Azores Archipelago. *Anaerobe* 24, 25–31. doi:10.1016/j.anaerobe.2013.09.004.

Saviolli, J. Y., Vieira Cunha, M. P., Lopes Guerra, M. F., Irino, K., Catao-Dias, J. L., and de Carvalho, V. M. (2016). Free-Ranging Frigates (Fregata magnificens) of the Southeast Coast of Brazil Harbor Extraintestinal Pathogenic Escherichia coli Resistant to Antimicrobials. *PLoS One* 11. doi:10.1371/journal.pone.0148624.

Sayah, R. S., Kaneene, J. B., Johnson, Y., and Miller, R. (2005). Patterns of antimicrobial resistance observed in Escherichia coli isolates obtained from domestic- and wild-animal fecal samples, human septage, and surface water. *Appl. Environ. Microbiol.* 71, 1394–1404. doi:10.1128/AEM.71.3.1394-1404.2005.

Schaefer, A. M., Bossart, G. D., Harrington, T., Fair, P. A., McCarthy, P. J., and Reif, J. S. (2019). Temporal Changes in Antibiotic Resistance Among Bacteria Isolated from Common Bottlenose Dolphins (Tursiops truncatus) in the Indian River Lagoon, Florida, 2003-2015. *Aquat. Mamm.* 45, 533–542. doi:10.1578/AM.45.5.2019.533.

Schaefer, A. M., Goldstein, J. D., Reif, J. S., Fair, P. A., and Bossart, G. D. (2009). Antibiotic-resistant organisms cultured from atlantic bottlenose dolphins (tursiops truncatus) inhabiting estuarine waters of charleston, sc and Indian River Lagoon, FL. *Ecohealth* 6, 33–41. doi:10.1007/s10393-009-0221-5.

Schierack, P., Römer, A., Jores, J., Kaspar, H., Guenther, S., Filter, M., et al. (2009). Isolation and characterization of intestinal Escherichia coli clones from wild boars in Germany. *Appl. Environ. Microbiol.* 75, 695–702.

Semedo-Lemsaddek, T., Pedroso, N. M., Freire, D., Nunes, T., Tavares, L., Verdade, L. M., et al. (2018). Otter fecal enterococci as general indicators of antimicrobial resistance dissemination in aquatic environments. *Ecol. Indic.* 85, 1113–1120. doi:10.1016/j.ecolind.2017.11.029.

Semedo-Lemsaddek, T., Nobrega, C. S., Ribeiro, T., Pedroso, N. M., Sales-Luis, T., Lemsaddek, A., et al. (2013). Virulence traits and antibiotic resistance among enterococci isolated from Eurasian otter (Lutra lutra). *Vet. Microbiol.* 163, 378–382. doi:10.1016/j.vetmic.2012.12.032.

Sen, K., Berglund, T., Soares, M. A., Taheri, B., Ma, Y., Khalil, L., et al. (2019). Antibiotic Resistance of E. coli Isolated From a Constructed Wetland Dominated by a Crow Roost, With Emphasis on ESBL and AmpC Containing E. coli. *Front. Microbiol.* 10. doi:10.3389/fmicb.2019.01034.

Sherley, M., Gordon, D. M., and Collignon, P. J. (2000). Variations in antibiotic resistance profile in Enterobacteriaceae isolated from wild Australian mammals. *Environ. Microbiol.* 2, 620–631. doi:10.1046/j.1462-2920.2000.00145.x.

Silva, N., Igrejas, G., Figueiredo, N., Gonçalves, A., Radhouani, H., Rodrigues, J., et al. (2010). Molecular characterization of antimicrobial resistance in enterococci and Escherichia coli isolates from European wild rabbit (Oryctolagus cuniculus). *Sci. Total Environ.* 408, 4871–4876.

Silva, N., Igrejas, G., Rodrigues, P., Rodrigues, T., Goncalves, A., Felgar, A. C., et al. (2011). Molecular characterization of vancomycin-resistant enterococci and extended-spectrum beta-lactamase-containing Escherichia coli isolates in wild birds from the Azores Archipelago. *AVIAN Pathol.* 40, 473–479. doi:10.1080/03079457.2011.599061.

Skurnik, D., Ruimy, R., Andremont, A., Amorin, C., Rouquet, P., Picard, B., et al. (2006). Effect of human vicinity on antimicrobial resistance and integrons in animal faecal Escherichia coli. *J. Antimicrob. Chemother.* 57, 1215–1219.

Smith, H. G., Clarke, R. H., Larkins, J.-A., Bean, D. C., and Greenhill, A. R. (2019). Wild Australian birds and drug-resistant bacteria: characterisation of antibiotic-resistant Escherichia coli and Enterococcus spp. *EMU-AUSTRAL Ornithol.* 119, 384–390. doi:10.1080/01584197.2019.1591162.

Smith, S., Wang, J., Fanning, S., and McMahon, B. J. (2014). Antimicrobial resistant bacteria in wild mammals and birds: a coincidence or cause for concern? *Ir. Vet. J.* 67. doi:10.1186/2046-0481-67-8.

Sousa, M., Goncalves, A., Silva, N., Serra, R., Alcaide, E., Zorrilla, I., et al. (2014). Acquired antibiotic resistance among wild animals: the case of Iberian Lynx (Lynx pardinus). *Vet. Q.* 34, 105–112. doi:10.1080/01652176.2014.949391.

Sousa, M., Silva, N., Igrejas, G., Sargo, R., Benito, D., Gomez, P., et al. (2016). Genetic Diversity and Antibiotic Resistance Among Coagulase-Negative Staphylococci Recovered from Birds of Prey in Portugal. *Microb. DRUG Resist.* 22, 727–730. doi:10.1089/mdr.2015.0266.

Sousa, M., Silva, N., Igrejas, G., Silva, F., Sargo, R., Alegria, N., et al. (2014). Antimicrobial resistance determinants in Staphylococcus spp. recovered from birds of prey in Portugal. *Vet. Microbiol.* 171, 436–440. doi:10.1016/j.vetmic.2014.02.034.

Souza, V., Rocha, M., Valera, A., and Eguiarte, L. E. (1999). Genetic Structure of Natural Populations of Escherichia coli in Wild Hosts on Different Continents. *Appl. Environ. Microbiol.* 65, 3373–3385.

Stedt, J., Bonnedahl, J., Hernandez, J., Waldenstrom, J., McMahon, B. J., Tolf, C., et al. (2015). Carriage of CTX-M type extended spectrum beta-lactamases (ESBLs) in gulls across Europe. *ACTA Vet. Scand.* 57. doi:10.1186/s13028-015-0166-3.

Stedt, J., Waldenstrom, J., Hernandez, J., Olsen, B., and Drobni, M. (2011). Divergent, and Locally High, Levels of Antibiotic Resistant Bacteria in European Herring Gulls (Larus argentatus) Conform to Patterns of Human Clinical Antibiotic Usage. *Ecohealth* 7, S98.

Stepien-Pysniak, D., Hauschild, T., Dec, M., Marek, A., and Urban-Chmiel, R. (2019). Clonal Structure and Antibiotic Resistance of Enterococcus spp. from Wild Birds in Poland. *Microb. DRUG Resist.* 25, 1227–1237. doi:10.1089/mdr.2018.0461.

Stepien-Pysniak, D., Hauschild, T., Nowaczek, A., Marek, A., and Dec, M. (2018). WILD BIRDS AS A POTENTIAL SOURCE OF KNOWN AND NOVEL MULTILOCUS SEQUENCE TYPES OF ANTIBIOTIC-RESISTANT ENTEROCOCCUS FAECALIS. *J. Wildl. Dis.* 54, 219–228. doi:10.7589/2017-05-118.

Stewart, J. R., Townsend, F. I., Lane, S. M., Dyar, E., Hohn, A. A., Rowles, T. K., et al. (2014). Survey of antibiotic-resistant bacteria isolated from bottlenose dolphins Tursiops truncatus in the southeastern USA. *Dis. Aquat. Organ.* 108, 91–102. doi:10.3354/dao02705.

Stoddard, R. A., Atwill, E. R., Gulland, F. M. D., Miller, M. A., Dabritz, H. A., Paradies, D. M., et al. (2008). Risk factors for infection with pathogenic and antimicrobial-resistant fecal bacteria in northern elephant seals in California. *Public Health Rep.* 123, 360–370.

Su, H., McKelvey, J., Rollins, D., Zhang, M., Brightsmith, D. J., Derr, J., et al. (2014). Cultivable Bacterial Microbiota of Northern Bobwhite (Colinus virginianus): A New Reservoir of Antimicrobial Resistance? *PLoS One* 9. doi:10.1371/journal.pone.0099826.

Sulzner, K., Kelly, T., Smith, W., and Johnson, C. K. (2014). ENTERIC PATHOGENS AND ANTIMICROBIAL RESISTANCE IN TURKEY VULTURES (CATHARTES AURA) FEEDING AT THE WILDLIFE-LIVESTOCK INTERFACE. *J. ZOO Wildl. Med.* 45, 931–934. doi:10.1638/2012-0217.1.

Swiecicka, I., Buczek, J., and Iwaniuk, A. (2003). Analysis of genetic relationships and antimicrobial susceptibility of Escherichia coli isolated from Clethrionomys glareolus. *J. Gen. Appl. Microbiol.* 49, 315–320.

Swift, B. M. C., Bennett, M., Waller, K., Dodd, C., Murray, A., Gomes, R. L., et al. (2019). Anthropogenic environmental drivers of antimicrobial resistance in wildlife. *Sci. Total Environ.* 649, 12–20. doi:10.1016/j.scitotenv.2018.08.180.

Szczepanska, B., Kaminski, P., Andrzejewska, M., Spica, D., Kartanas, E., Ulrich, W., et al. (2015). Prevalence, Virulence, and Antimicrobial Resistance of Campylobacter jejuni and Campylobacter coli in White Stork Ciconia ciconia in Poland. *FOODBORNE Pathog. Dis.* 12, 24–31. doi:10.1089/fpd.2014.1793.

Tausova, D., Dolejska, M., Cizek, A., Hanusova, L., Hrusakova, J., Svoboda, O., et al. (2012). Escherichia coli with extended-spectrum beta-lactamase and plasmid-mediated quinolone resistance genes in great cormorants and mallards in Central Europe. *J. Antimicrob. Chemother.* 67, 1103–1107. doi:10.1093/jac/dks017.

Thaller, M. C., Migliore, L., Marquez, C., Tapia, W., Cedeno, V., Rossolini, G. M., et al. (2010). Tracking Acquired Antibiotic Resistance in Commensal Bacteria of Galapagos Land Iguanas: No Man, No Resistance. *PLoS One* 5. doi:10.1371/journal.pone.0008989.

Thomas, M., Fenske, G. J., Antony, L., Ghimire, S., Welsh, R., Ramachandran, A., et al. (2017). Whole genome sequencing-based detection of antimicrobial resistance and virulence in non-typhoidal Salmonella enterica isolated from wildlife. *GUT Pathog.* 9. doi:10.1186/s13099-017-0213-x.

Troxler, S., Hess, C., Konicek, C., Knotek, Z., Bartak, P., and Hess, M. (2017). Microdilution testing reveals considerable and diverse antimicrobial resistance of Escherichia coli, thermophilic Campylobacter spp. and Salmonella spp. isolated from wild birds present in urban areas. *Eur. J. Wildl. Res.* 63. doi:10.1007/s10344-017-1125-2.

Van Breda, L. K., and Ward, M. P. (2017). Evidence of antimicrobial and disinfectant resistance in a remote, isolated wild pig population. *Prev. Vet. Med.* 147, 209–212. doi:10.1016/j.prevetmed.2017.09.014.

van de Giessen, A. W., van Santen-Verheuvel, M. G., Hengeveld, P. D., Bosch, T., Broens, E. M., and Reusken, C. B. E. M. E. M. (2009). Occurrence of methicillin-resistant Staphylococcus aureus in rats living on pig farms. *Prev. Vet. Med.* 91, 270–273. doi:10.1016/j.prevetmed.2009.05.016.

Vieira-da-Motta, O., Eckhardt-de-Pontes, L. A., Petrucci, M. P., dos Santos, I. P., da Cunha, I. C., and Morato, R. G. (2013). Microbiota and anthropic interference on antimicrobial resistance profile of bacteria isolated from Brazilian maned-wolf (Chrysocyon brachyurus). *BRAZILIAN J. Microbiol.* 44, 1321–1326.

Viswanathan, M., Pearl, D. L., Taboada, E. N., Parmley, E. J., Mutschall, S., and Jardine, C. M. (2017). Molecular and Statistical Analysis of Campylobacter spp. and Antimicrobial-Resistant Campylobacter Carriage in Wildlife and Livestock from Ontario Farms. *Zoonoses Public Health* 64, 194–203. doi:10.1111/zph.12295.

Vogt, N. A., Pearl, D. L., Taboada, E. N., Mutschall, S. K., Janecko, N., Reid-Smith, R. J., et al. (2019). Carriage of Campylobacter, Salmonella, and Antimicrobial-Resistant, Nonspecific Escherichia coli by Waterfowl Species Collected from Three Sources in Southern Ontario, Canada. *J. Wildl. Dis.* 55, 917–922. doi:10.7589/2018-12-288.

Vogt, N. A., Pearl, D. L., Taboada, E. N., Mutschall, S. K., Janecko, N., Reid-Smith, R., et al. (2018). Epidemiology of Campylobacter, Salmonella and antimicrobial resistant Escherichia coli in free-living Canada geese (Branta canadensis) from three sources in southern Ontario. *Zoonoses Public Health* 65, 873–886. doi:10.1111/zph.12511.

Vogt, N. A., Pearl, D. L., Taboada, E. N., Reid-Smith, R. J., Mulvey, M. R., Janecko, N., et al. (2019). A repeated cross-sectional study of the epidemiology of Campylobacter and antimicrobial resistant Enterobacteriaceae in free-living Canada geese in Guelph, Ontario, Canada. *Zoonoses Public Health* 66, 60–72. doi:10.1111/zph.12529.

Vredenburg, J., Varela, A. R., Hasan, B., Bertilsson, S., Olsen, B., Narciso-da-Rocha, C., et al. (2014). Quinolone-resistant Escherichia coli isolated from birds of prey in Portugal are genetically distinct from those isolated from water environments and gulls in Portugal, Spain and Sweden. *Environ. Microbiol.* 16, 995–1004. doi:10.1111/1462-2920.12231.

Waldenstrom, J., Mevius, D., Veldman, K., Broman, T., Hasselquist, D., and Olsen, B. (2005). Antimicrobial resistance profiles of Campylobacter jejuni isolates from wild birds in Sweden. *Appl. Environ. Microbiol.* 71, 2438–2441. doi:10.1128/AEM.71.5.2438-2441.2005.

Wallace, C. C., Yund, P. O., Ford, T. E., Matassa, K. A., and Bass, A. L. (2013). Increase in Antimicrobial Resistance in Bacteria Isolated from Stranded Marine Mammals of the Northwest Atlantic. *Ecohealth* 10, 201–210. doi:10.1007/s10393-013-0842-6.

Wang, J., Wong, E. S. W., Whitley, J. C., Li, J., Stringer, J. M., Short, K. R., et al. (2011). Ancient Antimicrobial Peptides Kill Antibiotic-Resistant Pathogens: Australian Mammals Provide New Options. *PLoS One* 6. doi:10.1371/journal.pone.0024030.

Wardyn, S.E., Kauffman, L.K. and Smith, T.C. (2012). Methicillin-resistant Staphylococcus aureus in central Iowa wildlife. *J. Wild. Dis.*, 48(4), pp.1069-1073.

Wasyl, D., Zajac, M., Lalak, A., Skarzynska, M., Samcik, I., Kwit, R., et al. (2018). Antimicrobial Resistance in Escherichia coli Isolated from Wild Animals in Poland. *Microb. DRUG Resist.* 24, 807–815. doi:10.1089/mdr.2017.0148.

Wheeler, E., Hong, P.-Y., Bedon, L. C., and Mackie, R. I. (2012). CARRIAGE OF ANTIBIOTIC-RESISTANT ENTERIC BACTERIA VARIES AMONG SITES IN GALAPAGOS REPTILES. *J. Wildl. Dis.* 48, 56–67.

WHITE, F. H., and FORRESTER, D. J. (1979). Anti-microbial resistant salmonella spp isolated from double-crested cormorants (Phalacrocorax auritus) and common loons (Gavia immer) in Florida. *J. Wildl. Dis.* 15, 235–237. doi:10.7589/0090-3558-15.2.235.

Williams, N. J., Sherlock, C., Jones, T. R., Clough, H. E., Telfer, S. E., Begon, M., et al. (2011). The prevalence of antimicrobial-resistant Escherichia coli in sympatric wild rodents varies by season and host. *J. Appl. Microbiol.* 110, 962–970. doi:10.1111/j.1365-2672.2011.04952.x.

Wu, J., Huang, Y., Rao, D., Zhang, Y., and Yang, K. (2018). Evidence for Environmental Dissemination of Antibiotic Resistance Mediated by Wild Birds. *Front. Microbiol.* 9. doi:10.3389/fmicb.2018.00745.

Yong, L. H., Ambu, S., Devi, S., and Maung, M. (2008). Detection of protozoan and bacterial pathogens of public health importance in faeces of Corvus spp. (large-billed crow). *Trop. Biomed.* 25, 134–139.

Zhang, A. Y., Wang, H. N., Tian, G. B., Zhang, Y., Yang, X., Xia, Q. Q., et al. (2009). Phenotypic and genotypic characterisation of antimicrobial resistance in faecal bacteria from 30 Giant pandas. *Int. J. Antimicrob. Agents* 33, 456–460. doi:10.1016/j.ijantimicag.2008.10.030.

Zhang, H., Zhao, X., Wang, X., and Chang, W. (2017). Prevalence and antimicrobial resistance profiles of Escherichia coli isolated from free-range pigs. *J. Infect. Dev. Ctries.* 11, 652–655. doi:10.3855/jidc.9269.

Zhang, M., Shen, Z., Rollins, D., Fales, W., and Zhang, S. (2017). Pilot Study of Antimicrobial Resistance in Northern Bobwhites (Colinus virginianus). *AVIAN Dis.* 61, 391–396. doi:10.1637/11629-031517-RegR.
